# Metabolic changes preceding bladder cancer occurrence among Korean men: a nested case-control study from the KCPS-II cohort

**DOI:** 10.1186/s40170-023-00324-0

**Published:** 2023-12-05

**Authors:** Youngmin Han, Unchong Kim, Keum Ji Jung, Ji-Young Lee, Kwangbae Lee, Sang Yop Shin, Heejin Kimm, Sun Ha Jee

**Affiliations:** 1 Institute for Health Promotion, Graduate School of Public Health, Yonsei University, Seoul, 03722 Republic of Korea; 2 Korea Medical Institute, Seoul, Republic of Korea

**Keywords:** Bladder cancer, Predictive biomarker, LC/MS metabolomics, Gaussian graphical model, Lysine metabolism, Tryptophan-indole metabolism

## Abstract

**Background:**

Bladder cancer (BLCA) research in Koreans is still lacking, especially in focusing on the prediction of BLCA. The current study aimed to discover metabolic signatures related to BLCA onset and confirm its potential as a biomarker.

**Methods:**

We designed two nested case-control studies using Korean Cancer Prevention Study (KCPS)-II. Only males aged 35–69 were randomly selected and divided into two sets by recruitment organizations [set 1, BLCA (*n* = 35) *vs.* control (*n* = 35); set 2, BLCA (*n* = 31) *vs.* control (*n* = 31)]. Baseline serum samples were analyzed by non-targeted metabolomics profiling, and OPLS-DA and network analysis were performed. Calculated genetic risk score (GRS) for BLCA from all KCPS participants was utilized for interpreting metabolomics data.

**Results:**

Critical metabolic signatures shown in the BLCA group were dysregulation of lysine metabolism and tryptophan-indole metabolism. Furthermore, the prediction model consisting of metabolites (lysine, tryptophan, indole, indoleacrylic acid, and indoleacetaldehyde) reflecting these metabolic signatures showed mighty BLCA predictive power (*AUC*: 0.959 [0.929–0.989]). The results of metabolic differences between GRS-high and GRS-low groups in BLCA indicated that the pathogenesis of BLCA is associated with a genetic predisposition. Besides, the predictive ability for BLCA on the model using GRS and five significant metabolites was powerful (*AUC*: 0.990 [0.980–1.000]).

**Conclusion:**

Metabolic signatures shown in the present research may be closely associated with BLCA pathogenesis. Metabolites involved in these could be predictive biomarkers for BLCA. It could be utilized for early diagnosis, prognostic diagnosis, and therapeutic targets for BLCA.

**Supplementary Information:**

The online version contains supplementary material available at 10.1186/s40170-023-00324-0.

## Introduction

Bladder cancer (BLCA) is a malignant tumor that develops in the bladder. The incidence rate of BLCA is increasing worldwide, and a poor prognosis has been reported when the tumor metastasizes [1, 2]; therefore, it is crucial to predict and manage the risk of BLCA occurrence.

The association of BLCA with many lifestyle risk factors (e.g., smoking and exposure to chemicals) has been established by numerous research [3, 4]. Xiong et al. [5] revealed a causal potential of smoking with BLCA using Mendelian randomization (MR). The odds ratio of BLCA for one SD increase in the number of cigarettes per day, lifetime smoking index, and smoking initiation indicated significance. The link between coffee consumption and BLCA is still unclear. A recent two-sample MR study showed no evidence that routine coffee consumption increased the incidence of BLCA [6]. Multiple observational studies have shown inconsistent results regarding the association between coffee consumption and BLCA [7–9]. This inconsistency in results could result from a complex interaction of several lifestyle factors.

Metabolomics, a comprehensive analysis of metabolites in biological samples, is an effective tool for exploring pathogenesis or discovering disease diagnostic markers. Changes in the metabolite level could be detected even before cancer diagnosis because metabolites closely reflect phenotype, including biochemical activity and the state of cells and tissues. There is growing evidence of the usefulness of metabolites as disease markers [10]. Several studies aiming to discover BLCA biomarkers using the metabolomics approach have been reported globally; mainly, urinary metabolites have been focused on BLCA because cancer cells and tissues are close to the urinary excretion route [11, 12].

Circulating metabolites in other specimens could also be helpful when screening high-risk BLCA groups. Indeed, Bansal et al. [13] observed serum biomarkers distinguished 81% of BLCA cases compared to healthy controls. The other metabolomics research revealed altered metabolic pathways of aromatic amino acids, glycolysis, citrate cycle, and lipogenesis metabolism in BLCA patients [14]. Serum substances, mainly glycans and lipids, are reported on comparisons based on the grades/stages of BLCA [15, 16]. Gupta et al. [17] identified significant serum metabolites relevant to BLCA at pre- and postoperative evaluation. Dimethylamine, lactate, histidine, and valine concentrations steadily decreased in both high- and low-grade BLCA patients at postoperative points; these metabolites exhibited a higher accuracy rate than urine cytology. In light of these findings, using serum metabolites for BLCA diagnostics may be a valuable alternative to or addition to urine cytology.

Gaussian graphical model (GGM) is an undirected visual model based on the partial correlation between two variables conditioned against the correlations with all other variables. Using partial correlation coefficients, GGM reduces the possibility of discovering spurious relationships. Partial correlations have recently been applied to biological data sets to interpret the network [18, 19].

Here, we expected to find serum biomarkers for predicting BLCA and provide insight into BLCA onset. Moreover, levels of circulating metabolites could be helpful to investigate the association between lifestyle factors and BLCA. To achieve goals, we designed a nested case-control study using the Korean Cancer Prevention Study (KCPS)-II with an LC/MS metabolomics approach. Baseline serum samples were analyzed by non-targeted metabolomics profiling. Through the GGM generated from the obtained data, we discover metabolic signatures related to BLCA onset. The association between metabolome and genetic variation was also indirectly explored using the genetic risk score (GRS) for BLCA generated from the genome-wide study (GWAS) conducted for all KCPS-II participants.

## Materials and methods

### Study population

Study subjects were selected from the KCPS-II cohort (*n* = 156,701). Briefly, cohort subjects were recruited through 18 health promotion centers across South Korea. Hospital admission records, death registers, and National Cancer Center Registry data were collected during the follow-up [20]. Additionally, data on self-completion of a questionnaire, anthropometric measurements, and biochemical indicators were analyzed in 12-h fasting blood [20]. During the follow-up period, 196 of the KCPS-II cohort individuals developed BLCA. Among them, we selected subjects whose serum conditions were appropriate for metabolite analysis.

Two independent sets were prepared to justify the metabolite results. Only males aged 35–69 and recruited through the university hospital (Seoul National Univ. Bundang Hosp., Yonsei Univ. Shinchon Severance and Gangnam Severance Hosp., Korean Univ. Guro Hosp., Ewha Womans Univ. Mokdong Hosp., CHA Univ. Bundang Cha Hosp.) and Korea Medical Institute (seven centers across South Korea) were included in the present study and divided by recruitment organizations (set 1, university hospital; set 2, Korea Medical Institute). Each set comprised BLCA and control groups at a ratio of 1:1 using propensity score matching (age and blood collection time point). The BLCA group had no cancer diagnosis at baseline (cohort enroll point) but was diagnosed with BLCA during the follow-up period; the mean time interval between baseline and BLCA onset was 7 years. In contrast, the control group remained free of all cancer, including BLCA, during the follow-up [set 1, BLCA (*n* = 35) *vs.* control (*n* = 35); set 2, BLCA (*n* = 31) *vs.* control (*n* = 31).

All procedures in the present research involving human participants were performed according to the ethical standards of the Institutional Review Board at the Yonsei University Health System under the Helsinki Declaration (IRB number: 4-2022-1136). Written informed consent was obtained from all subjects.

### Genome-wide association study and genetic risk score

DNA was genotyped using Affymetrix Genome-Wide Human SNP Array 5.0 (Affymetrix Inc.) at DNA Link Inc. (Seoul, Korea). Markers with a high missing rate (> 5%), individuals with a high missing rate (> 5%), and SNPs with minor allele frequency < 0.05 or in a significant deviation from Hardy-Weinberg equilibrium (*p* < 1.0E–6) were excluded for quality control. GWAS on BLCA was performed using PLINK 2.0. In order to estimate the effect size of each SNP for the BLCA, logistic regression was conducted by adjusting age. For construct GRS for BLCA, 92 SNPs with a *p* < 5 × 10^−5^ through Bonferroni correction from GWAS were selected (Table S1) and calculated as follows.

$${\textrm{GRS}}_{\textrm{i}}=\sum\nolimits_{j=1}^k Number\ of\ risk\ alleles\ in\ SNPj\times Weight,i=1,\cdots, n,\kern1em j=1,\cdots, k$$

n: total number of subjects, SNP_j_: jth SNP, Weight_j_: weight of the jth SNP

### Metabolome analysis

#### Non-targeted metabolomics

##### UHPLC-MS/MS analysis

Prepared serum samples were precipitated with cold acetonitrile (Wako Pure Chemical Industries, Osaka, Japan) (1:3, *v*/*v*) and centrifuged for 15 min (13,000 rpm, 4 °C). Separated supernatant was evaporated under nitrogen. Next, 100 μL of 10% methanol (J.T. Baker® Chemicals; Avantor Performance Materials, Inc., Radnor, PA, USA) dissolved the dried residue and filtrated with a 0.45-μm polyvinylidene difluoride syringe filter. L-Leucine-1-^13^C (Sigma-Aldrich, Saint Louis, MO, USA) was used as an internal standard (ISTD). The quality control (QC) sample was prepared following the exact step by combining all serum samples.

Serum samples were injected into the ACQUITY UPLC-BEH-C18 column (Waters, Milford, MA, USA) connected to the Thermo UHPLC System (UltiMate 3000 BioRS; Dionex, Thermo Fisher Scientific, Bremen, Germany). Compounds in samples were separated by a gradient system of the two mobile phases [A, 0.1% formic acid in LC-MS grade water (Thermo Fisher Scientific, Fair Lawn, NJ, USA); B, 0.1% formic acid in LC-MS grade methanol (Thermo Fisher Scientific, Fair Lawn, NJ, USA)] during 22 min. Q Exactive Plus Orbitrap (Thermo Fisher Scientific, Waltham, MA, USA) was combined with the UHPLC system for data detection. On MS, positive electrospray ionization mode (ESI+) was performed, and the data were collected in the full scan-ddms^2^ mode with a scan range of 80–1000 mass to charge (*m/z*). The MS conditions were set as follows: spray voltage, 3.0 kV; the flow rate of nitrogen sheath gas, 60 (arbitrary units); auxiliary gas, 20 (arbitrary units); capillary temperature, 370 °C; S-lens radiofrequency level, 45; and auxiliary gas heater temperature, 285 °C.

The QC sample was injected into every 10th sample for monitoring sensitivity and reproducibility during analysis. Apart from that, we measured QC samples repeatedly to assess the precision of our analyzing method. The intra-assay variation was estimated by analyzing seven replicates of QC samples in a day, and the inter-assay variation was calculated by the results of two different days. The precision of the intra- and inter-assay is presented as the relative standard deviation (%RSD).

##### Identification of metabolites

Compound Discoverer 3.0 software (Thermo Fisher Scientific, San Jose, CA, USA) was used for processing raw spectra. Initially, alignment (model, adaptive curve; maximum shift, 1 min; mass tolerance, 5 ppm) was conducted on selected spectra. Then, compounds were detected with an S/N threshold of 3 and mass tolerance of 5 ppm. After applying QC corrections and marking backgrounds, searching for annotation was followed. Dynamic recalibration was applied with an intensity tolerance of 30% and an intensity threshold of 0.1 for pattern/fragment matching under a mass tolerance of 5 ppm. Features detected less than 80% in all QC samples were discarded. Based on online databases [ChemSpider (http://www.chemspider.com), MassList (https://massbank.eu/MassBank/Search), Kyoto Encyclopedia of Genes and Genomes (KEGG; https://www.genome.jp/kegg), and mzCloud (https://www.mzcloud.org)], features were identified by formula, mass, and MS/MS data obtained in data-dependent acquisition mode (match factor threshold, 60).

### Statistical analysis

All statistical analyses were performed by R 4.1.3., except the principal component analysis (PCA), projection to latent structures-discriminant analysis (PLS-DA), orthogonal projection to latent structures-discriminant analysis (OPLS-DA), and metabolite set analysis. Independent *t*-tests and Mann–Whitney *U*-tests were used to evaluate the differences in clinical variables between the two groups. The distribution of variables was checked, and the skewed variables were logarithmically transformed. For nominal variables, a chi-square test was performed. The data are expressed as the mean ± SE, and two-tailed *p*-values less than 0.05 were considered to indicate significance.

For multivariate analyses, the normalized metabolite data were exported from Compound Discoverer 3.0 to MetaboAnalyst 5.0 (http://www.metaboanalyst.ca). After Pareto scaled and logarithmically transformed, PCA, PLS-DA, and OPLS-DA were performed. Metabolite set analysis using metabolites with significance (variable importance in the projection; *VIP* > 1.0) in both sets was conducted to evaluate whether predefined metabolic classes were related to BLCA.

Network analysis using GGM was created to visualize relationships between valuable metabolites revealed from our metabolite set analysis. We built GGM in Gaussian distribution based on conditional partial correlation, controlling the impact of additional metabolites except for two variables. Betweenness centrality was calculated by function centrality. To reflect the difference in quantitative metabolite abundance between BLCA and control groups, we calculated the z-score of each metabolite. At last, the ROC curve was created with metabolites showing significant results on network analysis for evaluating the predictive ability of BLCA.

## Results

### Anthropometric and clinical/biochemical characteristics at baseline

A summary of the overall baseline characteristics of subjects is presented in Table 1 and Table S2. There were no significant differences between the BLCA and control groups at baseline. After dividing subjects into the two sets, BMI [23.32 ± 0.47 (mean ± SE) in control *vs.* 24.73 ± 0.48 in BLCA; *p* = 0.043] and waist circumference [83.48 ± 1.23 in control *vs.* 86.76 ± 1.09 in BLCA; *p* = 0.045] were considerably higher in the BLCA group than in the control group in set 2. Except for these two indicators, the two groups had no significant differences. Even after adjusting BMI, there was no significant difference in clinical/biochemical variables between the two groups of set 2. In addition, among the total 66 BLCA subjects, 24 were transitional cell carcinoma, 32 were papillary carcinoma, and 4 were adenocarcinoma. There was no information about the remaining six people (Supplementary Figure 1).

**Table 1:** Baseline clinical and biochemical characteristics of subjects

	Set 1			Set 2			
	Total (*n* = 70)		*pa*	Total (*n*=62)		*pa*	*pb*
	Control (*n* = 35)	Bladder cancer occurrence (*n* = 35)		Control (*n* = 31)	Bladder cancer occurrence (*n* = 31)		
Age (year)	52.02 ± 1.10	54.51 ± 1.24	0.139	50.14 ± 1.78	50.26 ± 1.76	0.910†	
Current smoker *n*, (%)	13 (37.1)	13 (37.1)	0.737	14 (45.2)	19 (61.3)	0.112	
Body mass index (kg/m^2^)	25.19 ± 0.34	24.88 ± 0.44	0.666	23.32 ± 0.47	24.73 ± 0.48	0.043	
Waist circumference (cm)	87.73 ± 1.09	87.50 ± 1.23	0.919	83.48 ± 1.23	86.76 ± 1.09	0.045	0.476
Systolic blood pressure (mmHg)	125.40 ± 2.72	122.92 ± 2.68	0.507	123.71 ± 2.38	121.10 ± 2.56	0.528†	0.606
Diastolic blood pressure (mmHg)	81.69 ± 1.99	76.23 ± 1.76	0.041∮	77.89 ± 1.39	75.72 ± 1.46	0.302†	0.370
Glucose (mg/dL)	96.09 ± 2.44	98.17 ± 2.81	0.368†	96.82 ± 5.04	96.49 ± 5.59	0.578†	0.760
White blood cell (10^3^/μL)	6.03 ± 0.23	6.35 ± 0.24	0.251†	5.99 ± 0.27	6.52 ± 0.28	0.140∮	0.072
Albumin (g/dL)	4.64 ± 0.05	4.64 ± 0.04	0.920	4.45 ± 0.05	4.42 ± 0.04	0.704	0.467
Total cholesterol (mg/dL)	191.22 ± 6.38	195.61 ± 5.83	0.613	188.57 ± 6.62	201.73 ± 6.34	0.157	0.199
Triglyceride (mg/dL)	166.58 ± 15.33	148.42 ± 11.92	0.328∮	164.03 ± 16.87	170.30 ± 15.55	0.654∮	0.925
HDL cholesterol (mg/dL)	48.32 ± 1.86	48.01 ± 1.37	0.943∮	47.41 ± 1.26	47.67 ± 1.11	0.927†	0.508
LDL cholesterol (mg/dL)	114.50 ± 5.95	118.17 ± 5.33	0.639	112.82 ± 6.07	123.74 ± 6.37	0.216	0.260
AST (IU/L)	29.88 ± 3.94	24.31 ± 1.56	0.213†	33.28 ± 5.65	32.73 ± 6.98	0.921†	0.787
ALT (IU/L)	38.31 ± 9.52	28.30 ± 2.91	0.732†	34.02 ± 4.44	33.50 ± 4.09	0.693†	0.714
GGT (IU/L)	70.44 ± 16.93	44.82 ± 5.00	0.317†	86.03 ± 22.03	60.59 ± 8.52	0.632†	0.427
Bilirubin (mg/dL)	0.91 ± 0.07	0.95 ± 0.07	0.645∮	0.97 ± 0.08	0.88 ± 0.04	0.508†	0.636
Uric acid (mg/dL)	5.99 ± 0.27	6.10 ± 0.20	0.737	5.88 ± 0.23	6.00 ± 0.23	0.706	0.621
Blood urea nitrogen (mg/dL)	16.24 ± 1.77	14.87 ± 0.77	0.935†	15.18 ± 0.67	15.12 ± 0.66	0.949†	0.697
Creatinine (mg/dL)	1.25 ± 0.20	1.04 ± 0.03	0.999†	1.14 ± 0.03	1.08 ± 0.03	0.999†	0.079

Mean ± standard error (SE). Comparisons between the two groups (control *vs.* bladder cancer occurrence). Continuous variables were tested by an independent *t*-test, and variables marked with *∮* were tested by logarithmic transformation. Continuous variables with a nonnormal distribution even after logarithmic transformation were tested by a Mann-Whitney *U*-test, and *p*^*a*^-values are marked with *†*. *P*^*b*^-values are BMI-adjusted *p*^*a*^-values. Smoking status was tested by a chi-squared test. *AST* aspartate aminotransferase, *ALT* alanine aminotransferase, *GGT* γ-glutamyltransferase, *HDL* high-density lipoprotein, *LDL* low-density lipoprotein

Of the 66 BLCA subjects, 2 had no genetic analysis data; 64 were included in the analysis. The baseline characteristics of BLCA groups divided by GRS are shown in Table S3. The mean values of GRS were 39.5 (GRS-low group) and 53.6 (GRS-high group), respectively, and there was no significant difference in all variables except diastolic blood pressure (DBP) (*p* = 0.046).

### Discriminant metabolites between two groups

Our intra- and inter-assay precision data met the precision criteria and are presented in Table S4; *RSD* ≤ 15% is acceptable precision for LC-MS analysis [21]. Two-hundred fifty metabolites were identified among 2610 detected features. Multivariate statistical analysis was performed with these features.

The results of PCA and PLS-DA between BLCA and control groups are displayed in Supplementary Figure 2. In the PCA of set 1, the first three PCs (PC1, PC2, and PC3) accounted for approximately 72.3% of the total variance in the data. Similarly, the first three PCs accounted for about 77.7% in the set 2. Consequently, three PCs can collectively explain the variance in the model of both sets, describing the data distribution appropriately. There were comparable results in PLS-DA. The first three PCs accounted for around 78.4% in the PLS-DA of set 1 and 72.3% in set 2. Furthermore, the permutation test proved that both sets of models were statistically significant at *p* < 0.01 (0/100) level. The classification performance of PLS-DA was improved compared to PCA, as represented by the score plot.

**Fig. 1:**
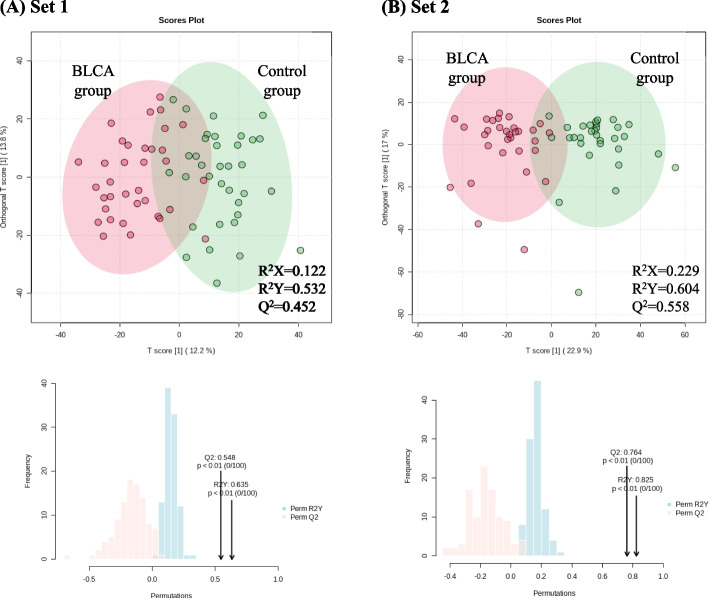
Identifying significant metabolites related to BLCA occurrence. **A** OPLS-DA score plots were obtained by comparison between the control (*n* = 35) and BLCA (*n* =35) groups of set 1. Permutation test set numbers: 100. **B** OPLS-DA score plots were obtained by comparison between the control (*n* = 31) and BLCA (*n* = 31) groups of set 2. Permutation test set numbers: 100

The results of OPLS-DA that maximize the visualization of each data contributing to discrimination by assigning a dependent variable are shown in Fig. 1. The validity of our models was assessed by the R^2^X, R^2^Y, and Q^2^ parameters. The model of both sets showed significance in evaluating prediction accuracy during 100 permutations [set 1, *Q*^2^ = 0.548 (*p* < 0.01), R^2^Y = 0.635 (*p* < 0.01); set 2, *Q*^2^ = 0.764 (*p* < 0.01), R^2^Y = 0.825 (*p* < 0.01)]. Comparison between the BLCA and control groups indicated significant differences (*VIP* > 1.0) in 93 metabolites in set 1 and 96 metabolites in set 2. Metabolites with *VIP* > 1.0 in both sets are listed in Table 2. Lysine predominantly influenced the distribution between the BLCA and control groups in both sets (VIP of set 1 = 2.150, VIP of set 2 = 1.891). Further, we conducted a metabolite set analysis on all subjects using significant metabolites listed in Table 2. As a result, we observed the superclass of organic acids, polyketides, benzoids, organic oxygen compounds, organoheterocyclic compounds, and fatty acyls were associated with BLCA risk at the *FDR* < 0.05 (Table S5).

**Table 2:** Non-targeted profiling of serum metabolites

Putative identification	HMBD ID	*m/z*	Formula	VIP		Fold change (BLCA/control)	
				Set 1	Set 2	Set 1	Set 2
Alanine	HMDB0000161	89.048	C_3_H_7_NO_2_	1.464	1.544	1.021	1.092
Toluene	HMDB0034168	92.063	C_7_H_8_	1.619	1.767	1.286	1.861
Benzaldehyde	HMDB0006115	106.042	C_7_H_6_O	1.229	1.820	1.333	1.857
Proline	HMDB0251528	115.063	C_5_H_9_NO_2_	1.467	1.641	1.125	0.977
Indole	HMDB0000738	117.058	C_8_H_7_N	1.470	1.661	1.325	1.779
Betaine	HMDB0000043	118.087	C_5_H_12_NO_2_	1.746	1.495	1.326	1.725
Threonine	HMDB0000167	119.058	C_4_H_9_NO_3_	1.799	1.477	1.365	1.735
Acetophenone	HMDB0033910	120.058	C_8_H_8_O	1.173	1.640	1.397	1.947
4-Hydroxybenzaldehyde	HMDB0011718	122.037	C_7_H_6_O_2_	1.022	1.695	1.301	1.937
Pipecolic acid	HMDB0000070	129.079	C_6_H_11_NO_2_	1.988	1.584	1.005	1.010
Glutaric acid	HMDB0000661	132.042	C_5_H_8_O_4_	1.052	1.367	1.691	1.543
Aspartic acid	HMDB0000191	133.038	C_4_H_7_NO_4_	1.467	1.061	1.478	1.704
Indole-3-carboxaldehyde	HMDB0029737	145.053	C_9_H_7_NO	1.397	1.673	1.000	0.991
Lysine	HMDB0000182	146.106	C_6_H_14_N_2_O_2_	2.150	1.891	1.395	1.862
Cinnamic acid	HMDB0000567	148.052	C_9_H_8_O_2_	1.784	1.830	1.281	1.883
Histidine	HMDB0000177	155.069	C_6_H_9_N_3_O_2_	1.140	1.745	1.230	1.789
Succinylacetone	HMDB0000635	158.058	C_7_H_10_O_4_	1.245	1.040	1.869	1.528
Indoleacetaldehyde	HMDB0001190	159.068	C_10_H_9_NO	1.365	1.634	1.320	1.743
Diethylmalonic acid	HMDB0251250	160.074	C_7_H_12_O_4_	1.217	1.308	1.435	0.921
Phenylalanine	HMDB0000159	165.079	C_9_H_11_NO_2_	1.804	1.113	1.284	1.836
Suberic acid	HMDB0000893	174.089	C_8_H_14_O_4_	1.260	1.523	1.894	1.810
Arginine	HMDB0000517	174.112	C_6_H_14_N_4_O_2_	1.726	1.571	1.474	1.954
Indoleacrylic acid	HMDB0000734	187.063	C_11_H_9_NO_2_	1.478	1.675	1.331	1.768
Tryptophan	HMDB0000929	204.090	C_11_H_12_N_2_O_2_	1.471	1.675	1.332	1.768
3-Hydroxysebacic acid	HMDB0000350	218.115	C_10_H_18_O_5_	1.080	1.828	1.580	1.789
Pantothenic acid	HMDB0000210	219.111	C_9_H_17_NO_5_	1.281	1.302	0.942	0.924
Biotin	HMDB0000030	244.088	C_10_H_16_N_2_O_3_S	1.171	1.271	2.806	1.757
Aspartyl-leucine	HMDB0028757	246.122	C_10_H_18_N_2_O_5_	1.537	1.131	1.501	1.353
Palmitoleamide	HMDB0256086	253.241	C_16_H_31_NO	1.138	1.612	1.192	0.938
*O*-Succinylcarnitine	HMDB0255869	261.121	C_11_H_19_NO_6_	1.513	1.293	1.747	1.051
Dibutylphthalic acid	HMDB0251177	278.152	C_16_H_22_O_4_	1.015	1.336	1.004	1.106
Diisobutyl phthalate	HMDB0013835	278.152	C_16_H_22_O_4_	1.309	1.364	1.062	0.997
6-Hydroxypentadecanedioic acid	HMDB0031885	288.194	C_15_H_28_O_5_	1.146	1.118	0.620	0.972
Palmitoylethanolamide	HMDB0002100	299.282	C_18_H_37_NO_2_	1.347	1.440	1.371	1.976
Sphinganine	HMDB0000269	301.298	C_18_H_39_NO_2_	1.237	1.034	0.954	1.139
Phytosphingosine	HMDB0004610	317.293	C_18_H_39_NO_3_	1.608	1.295	1.134	1.143
Oleoylethanolamide	HMDB0002088	325.298	C_20_H_39_NO_2_	1.085	1.224	1.033	2.055
Phenylalanyltyrosine	HMDB0256384	328.142	C_18_H_20_N_2_O_4_	1.154	1.142	1.963	0.749
Norethindrone acetate	HMDB0255731	340.204	C_22_H_28_O_3_	1.633	1.098	1.414	1.441
11*β*-Prostaglandin F2α	HMDB0010199	354.241	C_20_H_34_O_5_	1.705	1.058	1.816	1.072
1-Stearoylglycerol	HMDB0244009	358.308	C_21_H_42_O_4_	1.213	1.560	0.970	1.016
Furanylfentanyl	HMDB0259455	374.199	C_24_H_26_N_2_O_2_	1.742	1.005	2.464	1.592
LysoPC(14:0/0:0)	HMDB0010379	467.301	C_22_H_46_NO_7_P	1.172	1.051	1.138	1.055
LysoPC(P-16:0/0:0)	HMDB0010407	479.338	C_24_H_50_NO_6_P	1.654	1.161	1.475	0.867
LysoPC(15:0/0:0)	HMDB0010381	481.317	C_23_H_48_NO_7_P	1.506	1.207	1.367	1.021
LysoPE(20:0/0:0)	HMDB0011511	509.348	C_25_H_52_NO_7_P	1.597	1.473	1.352	1.058
LysoPC(20:5/0:0)	HMDB0010397	541.317	C_28_H_48_NO_7_P	1.005	1.009	1.138	1.056
LysoPC(20:0/0:0)	HMDB0010390	551.395	C_28_H_58_NO_7_P	1.223	1.446	1.680	0.985
Sphingomyelin(d18:1/14:1)	HMDB0240612	672.520	C_37_H_73_N_2_O_6_P	1.104	1.607	1.100	1.031

*LysoPC* lysophosphatidylcholine, *LysoPE* lysophosphatidylethanolamine. Metabolites with variable importance in the projection (VIP) values > 1.0 in both sets are listed in Table 2. VIP value was obtained from orthogonal projection to latent structures discriminant analysis (OPLS-DA), analyzing discriminant metabolites between BLCA and control groups. Fold change (BLCA/control) was calculated using the relative abundance of each metabolite

In principal component analysis (PCA), PLS-DA, and OPLS-DA analyses between the morphology groups of BLCA patients (Supplementary Figure 1), no metabolites represented appreciable differences between transitional cell carcinoma, papillary carcinoma, and adenocarcinoma. The variations between the two groups, except for adenocarcinoma, which had a small sample size, were not accounted for by metabolites (Supplementary Figure 1).

### Metabolite network analysis

We created GGM based on z-score, betweenness centrality, and partial correlation to explore the critical network between selected 23 metabolites on metabolite set analysis (Table S5). GGM comprises a set of metabolites depicted by edge and node visualizing relationships between them. Betweenness centrality was calculated with a cutoff of 0.5.

**Fig. 2:**
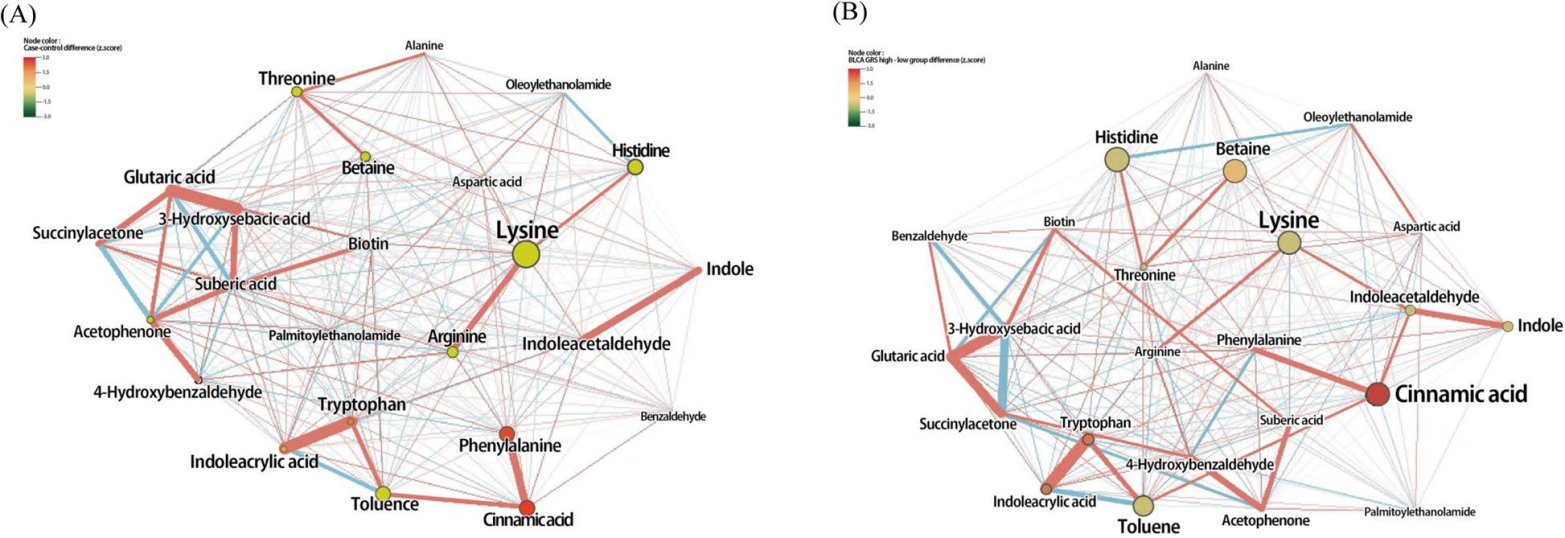
GGM network with significant metabolites related to BLCA occurrence. Node presents a metabolite, and the edge between two nodes means a conditional partial correlation. The size and color of nodes represent betweenness centrality and effect sizes, respectively. The nodes with significance (*FDR* < 0.05) are outlined in gray. Positive and negative correlations are represented using light red and light blue edges, respectively. Thicker edges represent stronger correlations between two metabolite levels. **A** The network between the control (*n* = 66) and BLCA (*n* = 66) groups. **B** The network between the GRS low-BLCA (*n* = 32) and GRS high-BLCA (*n* = 32) groups

First, we created a GGM network between BLCA and control groups (Fig. 2A). Seventeen were different in z-score at *FDR* < 0.05 threshold (betaine, phenylalanine, alanine, threonine, histidine, lysine, aspartic acid, arginine, tryptophan, cinnamic acid, toluene, benzaldehyde, 4-hydroxybenzaldehyde, acetophenone, indoleacrylic acid, indole, and indoleacetaldehyde). The main networks are summarized as follows. Lysine, which had the highest VIP value in both two independent sets and the highest betweenness centrality of 34.5, correlated positively with histidine (*r* = 0.222, *p* = 0.001), arginine (*r* = 0.442, *p* < 0.001), and alanine (*r* = 0.293, *p* = 0.047). The next highest centrality was 6.34, which corresponded to toluene, cinnamic acid, histidine, and phenylalanine. Several significant networks were also identified between these metabolites. Several strong correlations (|*r*| > 0.5, *p* < 0.001), including these metabolites, were summarized: toluene and indoleacrylic acid (*r* = −0.508), toluene and tryptophan (*r* = 0.512), indole and indoleacetaldehyde (*r* = 0.594), and cinnamic acid and phenylalanine (*r* = 0.589).

As a result of network analysis between two BLCA groups divided by GRS for BLCA (Fig. 2B), metabolites of significant difference on z-score at *FDR* < 0.05 threshold were the same as the GGM network between BLCA and control groups. Among them, lysine still indicated high betweenness centrality of 24.5. Lysine showed positive correlations with indoleacetaldehyde (*r* = 0.359, *p* = 0.020) and arginine (*r* = 0.442, *p* = 0.003). Cinnamic acid, betweenness centrality of 24.3, positively correlated with toluene (*r* = 0.366, *p* = 0.017); toluene showed a strong negative correlation with indoleacrylic acid (*r* = −0.508, *p* = 0.001) and positive correlation with tryptophan (*r* = 0.512, *p* = 0.001). Histidine, betweenness centrality of 23.8, showed a correlation with threonine (*r* = 0.397, *p* = 0.009). Interestingly, toluene and betaine indicated higher betweenness centrality compared to GGM between BLCA and control groups.

### Logistic regression analysis

**Fig. 3:**
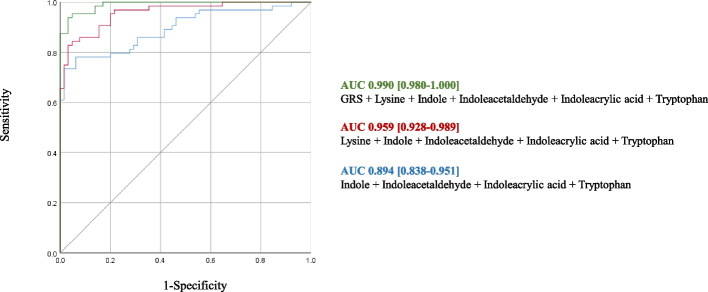
ROC curves for prediction of BLCA. Prediction models in the total subjects (*n* = 132). The green line is a BLCA prediction model consisting of GRS, lysine, tryptophan, indole, indoleacrylic acid, and indoleacetaldehyde. The red line is a BLCA prediction model consisting of lysine, tryptophan, indole, indoleacrylic acid, and indoleacetaldehyde. The blue line is a BLCA prediction model consisting of tryptophan, indole, indoleacrylic acid, and indoleacetaldehyde. The gray line is a reference line

Figure 3A shows the prediction model using significant biomarkers revealed in the present research. An area under the curve (AUC) obtained from the prediction model consisting of lysine, tryptophan, indole, indoleacrylic acid, and indoleacetaldehyde was 0.959 (95% *CI*: 0.929–0.989). An AUC of 0.899 (95% *CI*: 0.844–0.953] was observed in the predictive model consisting of four novel metabolites, except for lysine, which has been reported as a BLCA-related metabolite. Therefore, these metabolites are expected to be usefully utilized as biomarkers for BLCA risk diagnosis. As a result of the BLCA prediction model by adding GRS for BLCA to lysine, tryptophan, indole, indoleacrylic acid, and indoleacetaldehyde, an AUC was calculated as 0.990 (95% *CI*: 0.980–1.000).

### The relationship between smoking status and metabolites

We conducted a logistic regression analysis in BLCA to identify the independent risk factors of current smoking status (Table S6). Twenty-three metabolites used for GGM entered into multivariable logistic regression models. Two meaningful metabolites related to current smoking status were discovered; threonine (*OR* = 1.041, *p* = 0.024) and acetophenone (*OR* = 0.782, *p* = 0.025). However, threonine and acetophenone were not statistically different between smoking status (never *vs.* ever *vs.* current smoker) of BLCA subjects in the analysis of variance (data not shown).

## Discussion

We demonstrated metabolic changes preceding BLCA occurrence in Korean men via metabolomics technology. In particular, we selected replicated metabolites in two independent sets for further statistical analysis. In the finally drawn GGM model, we found that BLCA risk-related metabolic changes occurred simultaneously in multiple metabolomes and correlated with each other; further, they seemed to be associated with a genetic predisposition. Moreover, it is worth noting that these metabolic characteristics preceded the change in creatinine, one of the well-known indicators of BLCA.

The first notable is a metabolic pathway involving lysine. Lysine showed the highest VIP value in both two independent sets. In other words, lysine can explain the most remarkable metabolic difference between the BLCA and the control groups. Besides, it had significantly correlated with several meaningful metabolites. GGM delineated that lysine has the highest betweenness centrality value. Significant variations of lysine in BLCA have been found in several studies. Compared to European American (EA) BLCA serum, upregulated levels of lysine were seen in African American (AA) BLCA serum, which were not detectable in serum from healthy AA and EA controls; the result suggested that the metabolic abnormalities between AA and EA patients with BLCA were indicative of the disease [22]. However, a pilot urine analysis conducted by Kim et al. [23] revealed urinary lysine level was slightly lower in BLCA than in the control in the Korean population; the outcomes of Kim’s study may be inconsistent with the current study due to the considerable small sample size.

Lysine signatures observed in the present research could be from abnormal chromatin modulation. Chromatin remodeling is modulated by histone acetylation, methylation, phosphorylation, and ubiquitination on specific residues of lysine, arginine, and serine. Furthermore, acetyl-CoA is derived from branched-chain amino acids, and lysine is used for protein acetylation for building blocks of cancer cells [24]. These posttranslational modifications are closely related to cancer pathogenesis, regulating immune cell differentiation and activation [25]. Moreover, epigenetic alterations like chromatin remodeling gene mutation commonly appear in BLCA patients [26, 27]. Taken together, observed dysregulation of lysine-related metabolism in the BLCA is likely related to epigenetic alteration affecting chromatin modulation. Although this study could not prove a direct interaction, it is assumed that abnormal lysine metabolism is closely associated with genetic factors, considering that the betweenness centrality of lysine remained the highest in the analysis of divided BLCA groups into the GRS for BLCA. Confirming the association using functional annotation analysis of genetic data is necessary.

The next thing to note is the tryptophan-indole axis. In our results, tryptophan, indole, indoleacetaldehyde, and indoleacrylic acid were detected as metabolites related to this axis, indicating a significant correlation with each other; interactions among metabolites in different chemical super-class classifications were also observed. GGM revealed that this metabolic axis is related to lysine. This association was more robust when comparing BLCA groups divided by GRS for BLCA.

Regarding tryptophan, several studies are lined with our results; however, they discovered tryptophan as a urinary candidate biomarker in BLCA patients [28, 29]. In research from Ossolinski et al. [15], urinary tryptophan concentrations were higher in non-muscle-invasive BLCA patients, contributing to the separation between non-muscle-invasive and muscle-invasive BLCA patients, showing a VIP value of 1.7. However, the significance of urinary tryptophan was not demonstrated in the comparison of BLCA and controls in their study [15]. Meanwhile, Kim et al. [23] observed slightly decreased urinary levels of tryptophan in patients with BLCA compared with controls in their pilot study. In our study, BLCA groups had greater serum tryptophan concentrations than control groups. Similar findings to our data were reported by Alberice et al. [30], who found that serum tryptophan levels increased as BLCA progressed. In contrast, there was no discernible difference in serum tryptophan levels between non-muscle-invasive BLCA and healthy controls [31].

Tryptophan is metabolized through serotonin, kynurenine, and indole pathways. Evidence of dysregulated tryptophan-kynurenine axis in cancer has been reported [32, 33]; the key is aryl hydrocarbon receptor (AhR), a transcription factor modulating cytochrome P450s. Especially in the tryptophan-kynurenine axis, indoleamine 2,3-dioxygenase-1 (IDO1) acts as critical immunomodulator of AhR. IDO1 is the rate-limiting enzyme in the degradation of tryptophan, and catabolites produced by it activate AhR. Furthermore, Tsai et al. [34] revealed that IDO1 expression in urothelial carcinoma tissue predicts poorer survival in BLCA. In other words, AhR expression is abundant in human tumor cells and closely related to the immune system and function. Our results give insight into possible mechanisms through indole derivates from tryptophan to cause carcinogenesis in the bladder via AhR.

The intestinal bacteria converts tryptophan mostly into indole by the bacterial enzyme tryptophanase and can also convert tryptophan directly or indirectly into indole derivates, including indole-3-acrylate and indole acetaldehyde [35, 36]. However, the specific metabolic processes by which the intestinal microbiota converts tryptophan into these minor indolic chemicals are still unclear [36]. Indole derivates affect innate and adaptive immune responses as a ligand of AhR in intestinal immune cells, increasing the production of interleukin (IL)-22, which amplifies IL-1β release in adipose tissue macrophage via the C-Jun pathway [37]. Despite the lack of evidence regarding the association between BLCA and indole derivatives, several studies have documented that indole alkaloids play a role in regulated cell death, such as apoptosis, ferroptosis, and necroptosis controlling cell signaling [38, 39]. For example, harmaline, a classic indole alkaloid, has been explored for its ability to inhibit gastric tumor growth both in vitro and in vivo upregulating Fas/FasL expression and further activating caspase-8/-3 [40]. Carboline alkaloids could trigger human ovarian cancer cells’ apoptosis by activating caspase- and reactive oxygen species (ROS)-dependent pathways [41]. Besides, some indole-containing compounds are already in clinical application for treating various types of cancer, even drug-resistant cancer [38]. From these points, we suggested the tryptophan-indole axis is expected to be a new target for BLCA; further experimental studies are needed to elucidate the exact mechanism of the tryptophan-indole axis associated with BLCA. In addition, further studies on the relationship with genetic factors are required.

Recently, research regarding the genetic predisposition for BLCA has been reported. One of the emerging findings of genetic factors for BLCA is related to BP [42, 43]. In hypertensive individuals, an overactive renin-angiotensin system (RAS) increases the incidence and mortality of cancer [42]. In hypertensive conditions, chronic inflammation and overexpressed vascular endothelial growth factors contribute to endothelial dysfunction and angiogenesis, which are closely associated with the proliferation of cancer cells. In line with this, our study observed that BLCA with high GRS showed significantly higher DBP than the low GRS group. Therefore, the present research may provide crucial evidence for an association between BP and BLCA, which is rarely revealed in the Korean population. However, confirmation in studies with larger sample numbers is needed.

Finally, we discovered two meaningful metabolites associated with smoking. Although we could not elucidate where the metabolites come from, we confirmed the results supported by the literature review. A close association between BLCA and lifestyle risk factors such as diet, smoking, and environmental exposure has been established through numerous studies [44]. According to the human metabolome database (HMDB), acetophenone (HMDB ID: 0033910), the simplest aromatic ketone, has been reported as an ingredient for flavoring, cigarette additives, and some pharmaceuticals. Further, associations between acetophenone and ulcerative colitis and Crohn’s disease have been discovered. In our data, a negative association was observed between acetophenone and smoking status, although acetophenone showed increased levels in the BLCA group compared to the control group. Besides, the GGM network revealed significant metabolic traits involving acetophenone. A series of studies identified a positive correlation between smoking and cancer risk [5, 45]. Considering this, our result is unexpected, but it is not easy to confirm a direct relationship as smoking-related metabolic substances in the body were not considered together. Unfortunately, no literature could be found to support the relationship between threonine and smoking status shown in this study.

Our study has several limitations. First, only Korean men were included in the present study. Therefore, it is necessary to confirm these metabolic characteristics in other races and women. It was challenging to undertake replication analysis utilizing the UK Biobank (UKBB), a representative European ethnic cohort, in the current study. A review of papers on BLCA biomarkers using the UKBB [46–48] revealed no matching results on SNP identified in our investigation, and no BLCA-metabolome studies based on UKBB were found. Consequently, our findings could be confined to this population; further studies on other races are needed. Additionally, the small sample size is also a major limitation. Due to the small sample, establishing the difference of metabolites in the morphological classification of BLCA was also difficult. Next, drawing the causality of the metabolic signatures in the present study was challenging. Prospectively verifying metabolites that are associated with BLCA risk in this study with exploring the causal relationship between these metabolic signatures is needed. Third, the underlying mechanisms of the metabolic characteristics seen in the BLCA group could not be clearly explained. Since we suggested a possible metabolic mechanism through a literature review, additional experimental studies are needed to elucidate the exact mechanism of BLCA pathogenesis reflected by the metabolic signatures revealed in this study. To complement the weaknesses of our research, we performed functional validation using functional analysis mapping and annotation (FUMA) of the GWAS platform (https://fuma.ctglab.nl/). The gene ontology enrichment analysis results based on our GWAS data are summarized in Table S7; 23 gene sets contained several genes that matched SNPs at the *p* < 5 × 10^−5^ level from GWAS. However, in most cases, only one gene was matched, while two genes were linked in the following five pathways (lymphocyte activation, cell activation, peptidyl amino acid modification, protein phosphorylation, and phagocytosis). Although these seemed closely related to the metabolic pathways mentioned in the discussion, follow-up experimental research is needed to accurately verify the metabolite biomarkers our study discovered. Moreover, we tried to validate our results using external data. Gene ontology enrichment analysis using four Chinese male samples [BLCA (*n* = 2) vs. control (*n* = 2)] from Li et al. [49] was conducted on BioJupies (https://maayanlab.cloud/biojupies/). BLCA patients were a 70-year-old subject with T3N0M0 and a 58-year-old with T1(G3)N0M0 BLCA. The ages of the subjects included in the control group were 70 and 58 years, respectively, which were the same as those in the BLCA group. As a result, we identified several gene sets showing significance in the external set (Supplementary Figure 3). Among them, regulation of signal transduction contained two genes (*AXL* and *KANK1*) matched SNPs at the *p* < 5 × 10^−5^ level from our GWAS. Although there is a limitation in using external data with the small sample size, it is significant that results from RNA sequencing data of Asian men partially overlap with our study. However, external data with more samples are needed to prove the effectiveness of our model in the real world.

Despite these limitations, we revealed metabolic signatures already seen before the onset of BLCA using a large-scale prospective Korean cohort. To the best of our knowledge, this study is the first long-term metabolomics research for BLCA conducted in Korean. A major strength of the present study is that GGM network analysis was performed between significant metabolites repeatedly detected in two independent sets. Another strength is that the metabolomic data were interpreted together with the constructed GRS for BLCA. To summarize, we identified the dysregulation of lysine metabolism and tryptophan-indole metabolism as notable metabolic signatures of the BLCA group, and it is assumed that they are associated with a genetic predisposition. Besides, the predictive ability for BLCA on the model using GRS and five significant metabolites was powerful. These are likely a BLCA pathogenesis mechanism, and they could be used as predictive biomarkers of BLCA. Conclusionally, it could be a key to research on the BLCA, including early diagnosis, prognostic diagnosis, and therapeutic targets.

### Supplementary Information

**Additional file 1: Supplementary Figure 1.** The morphology distribution of BLCA patients (*n*=66) & Comparisons of groups divided by morphology. **Supplementary Figure 2.** Comparisons between BLCA and control groups. **Supplementary Figure 3.** External validation_Gene Ontology Enrichment Analysis Results

**Additional file 2.** **Supplementary Table S1.** SNPs used to construct GRS.

**Additional file 3.** **Supplementary Table S2.** Baseline clinical and biochemical characteristics of subjects (all).

**Additional file 4.** **Supplementary Table S3.** Baseline clinical and biochemical characteristics of BLCA subjects.

**Additional file 5.** **Supplementary Table S4.** LC/MS method validation.

**Additional file 6.** **Supplementary Table S5.** Metabolite set analysis for super-class associated with bladder cancer risk in all subjects.

**Additional file 7.** **Supplementary Table S6.** Logistic regression analysis to identify smoking-related metabolites in BLCA.

**Additional file 8.** **Supplementary Table S7.** Gene Ontology Enrichment Analysis Results of our data using FUMA.

## Data Availability

Some or all datasets generated during and/or analyzed during the current study are not publicly available but are available from the corresponding author upon reasonable request.

## Abbreviations

AhR: Aryl hydrocarbon receptor
AUC: Area under the curve
BLCA: Bladder cancer
DBP: Diastolic blood pressure
GGM: Gaussian graphical model
GRS: Genetic risk score
GWAS: Genome-wide study
HMDB: Human metabolome database
ISTD: Internal standard
KCPS: Korean Cancer Prevention Study
OPLS-DA: Orthogonal projection to latent structures-discriminant analysis
PCA: Principal component analysis
PLS-DA: Projection to latent structures-discriminant analysis
QC: Quality control
RAS: Renin-angiotensin system
VIP: Variable importance in the projection
