# Supplementary material for: Metabolic changes preceding bladder cancer occurrence among Korean men: a nested case-control study from the KCPS-II cohort
Source: Cancer Metab. 2023 Dec 5;11:23. doi: 10.1186/s40170-023-00324-0 (PMC10696702; doi:10.1186/s40170-023-00324-0)
Supplement: Supplementary file 1 — Additional file 1: Supplementary Figure 1. The morphology distribution of BLCA patients (n=66) & Comparisons of groups divided by morphology. Supplementary Figure 2. Comparisons between BLCA and control groups. Supplementary Figure 3. External validation_Gene Ontology Enrichment Analysis Results [file 40170_2023_324_MOESM1_ESM.pdf]

Supplementary Figure 1

The morphology distribution of BLCA patients (n=66)

ICD-O-3 Morphology Codes	Transitional cell carcinoma<br>81203	Papillary carcinoma<br>81303	Adenocarcinoma<br>81403	Unknown
N (%)	24 (36.4)	32 (48.5)	4 (6.1)	6 (9.1)

Comparisons of groups divided by morphology

(A) Transitional cell carcinoma *vs.* Papillary carcinoma *vs.* Adenocarcinoma

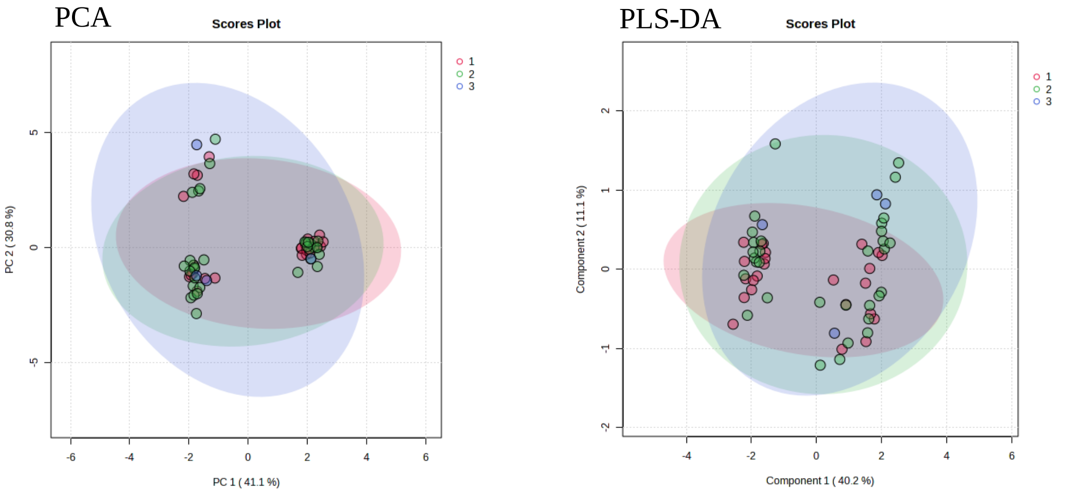

(B) Transitional cell carcinoma vs. Papillary carcinoma

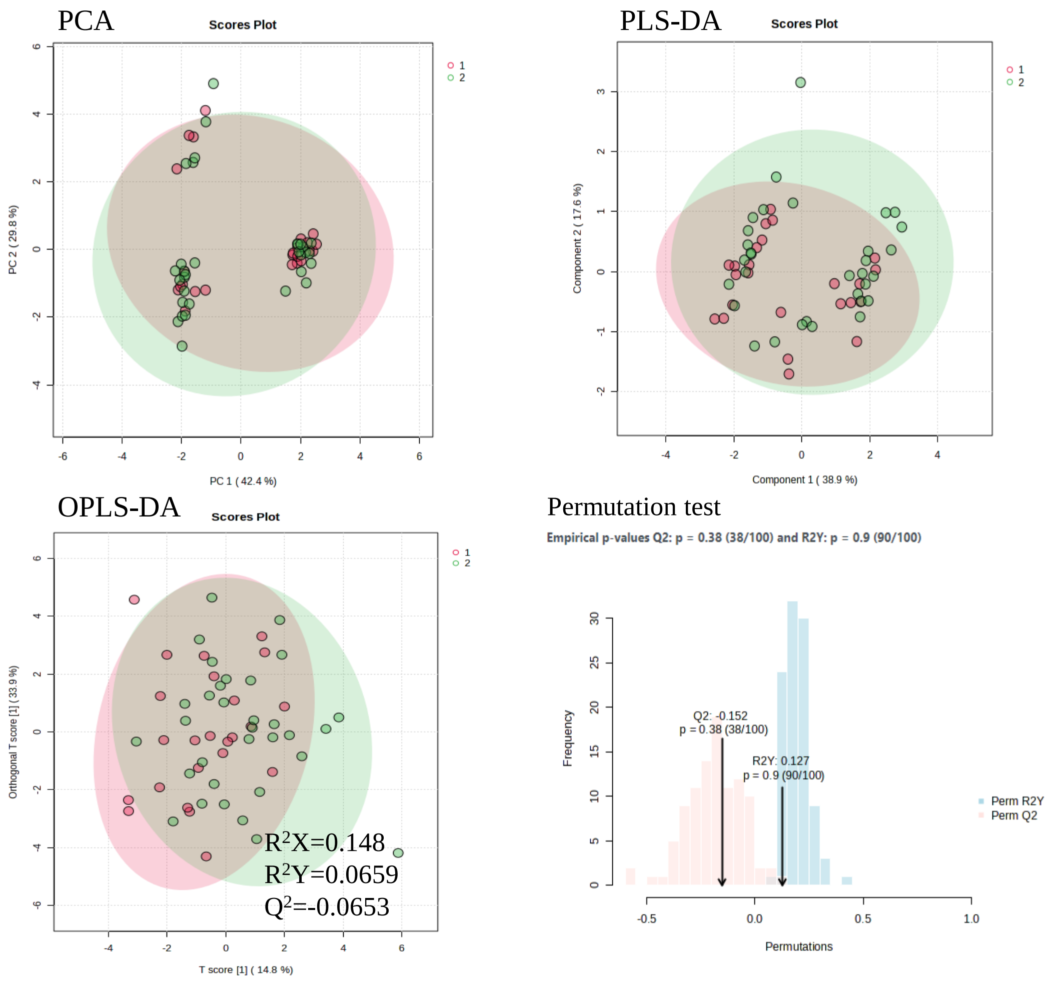

Supplementary Figure 2

Comparisons between BLCA and control groups

(A) PCA

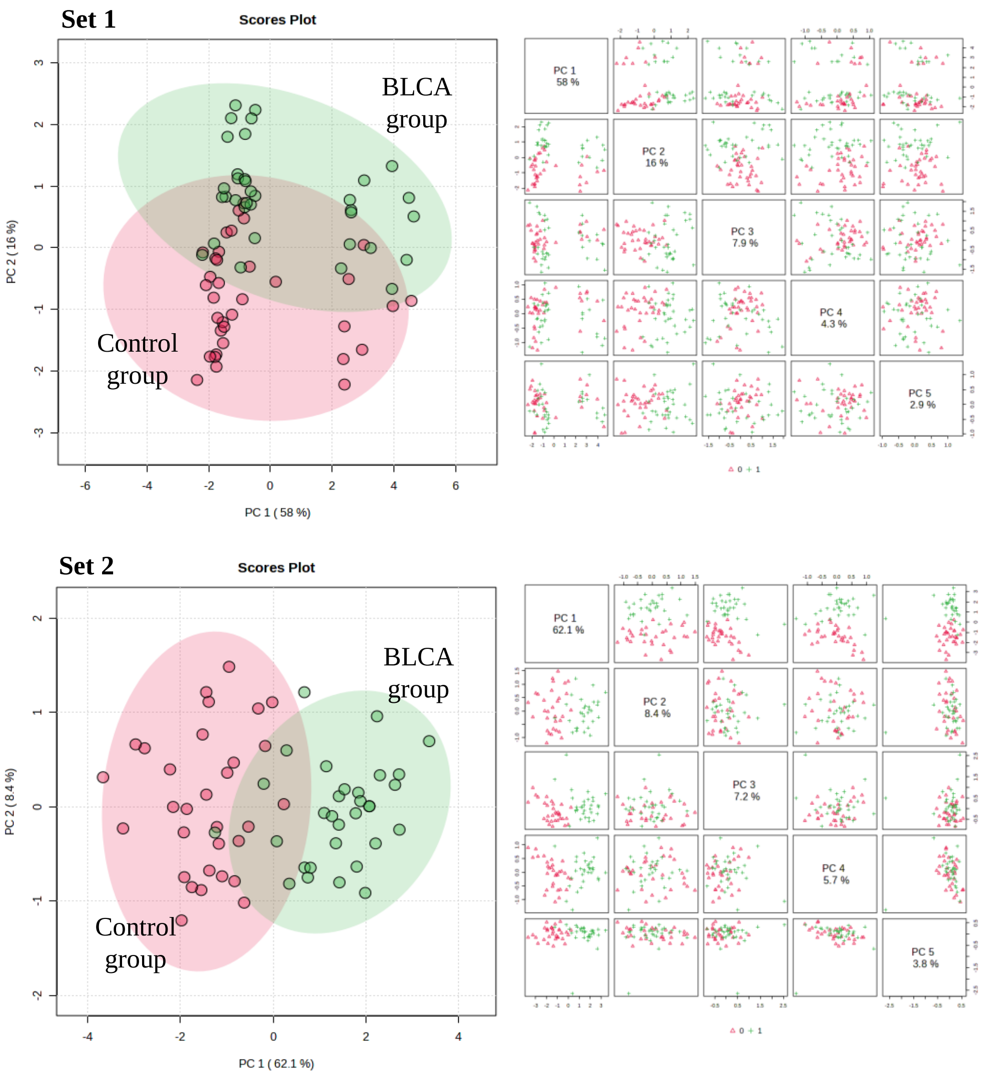

(B) PLS-DA

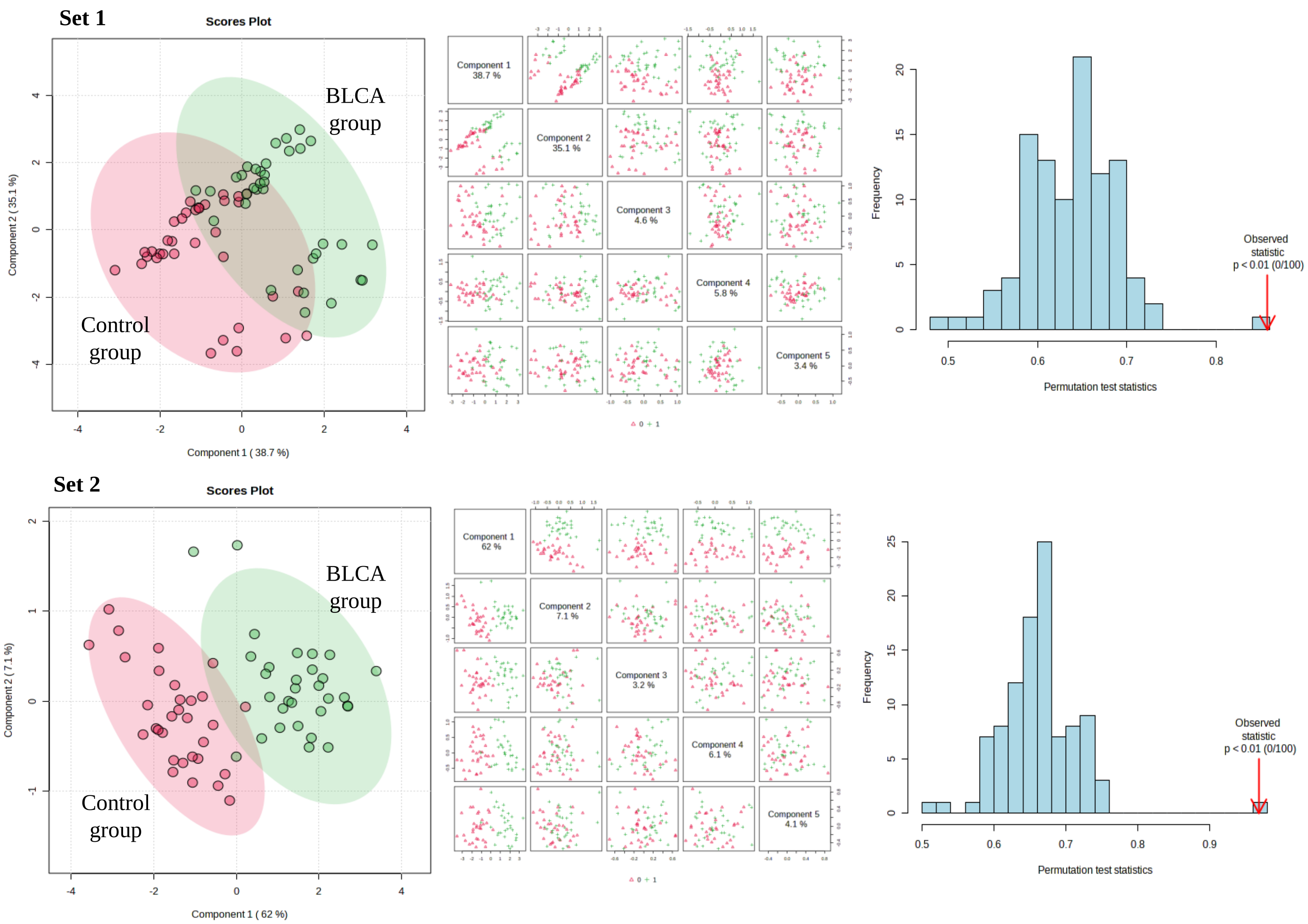

Supplementary Figure 3

External validation_ Gene Ontology Enrichment Analysis Results

Control (*n* = 2) *vs.* BLCA (*n* = 2)_Gene Ontology Biological Process

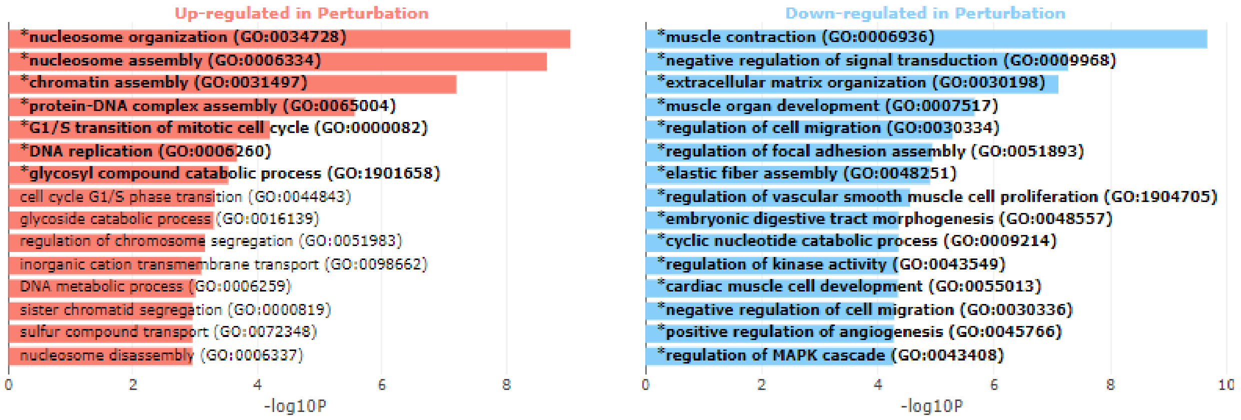

Control (*n* = 2) *vs.* BLCA (*n* = 2)_Gene Ontology Cellular Component

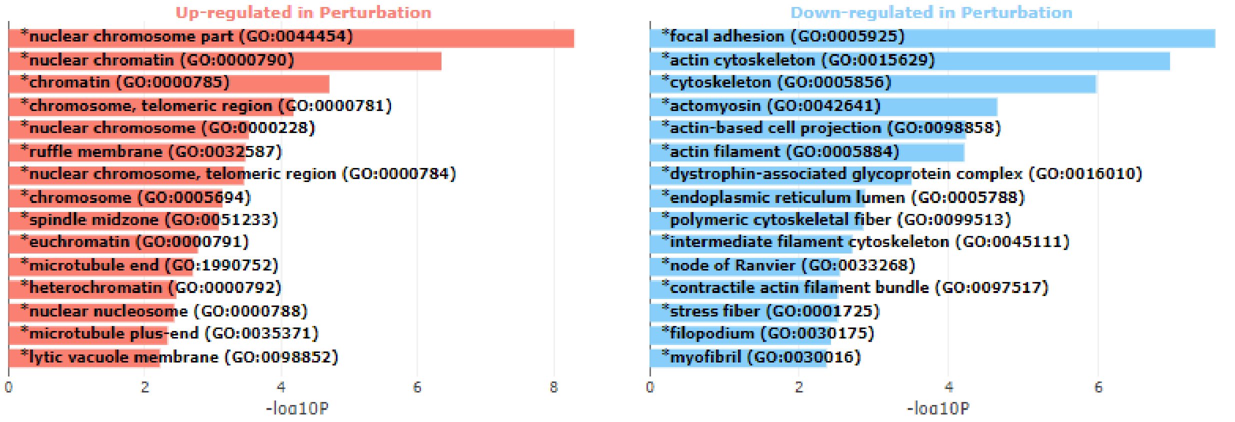

Control (*n* = 2) *vs.* BLCA (*n* = 2)_Gene Ontology Molecular Function

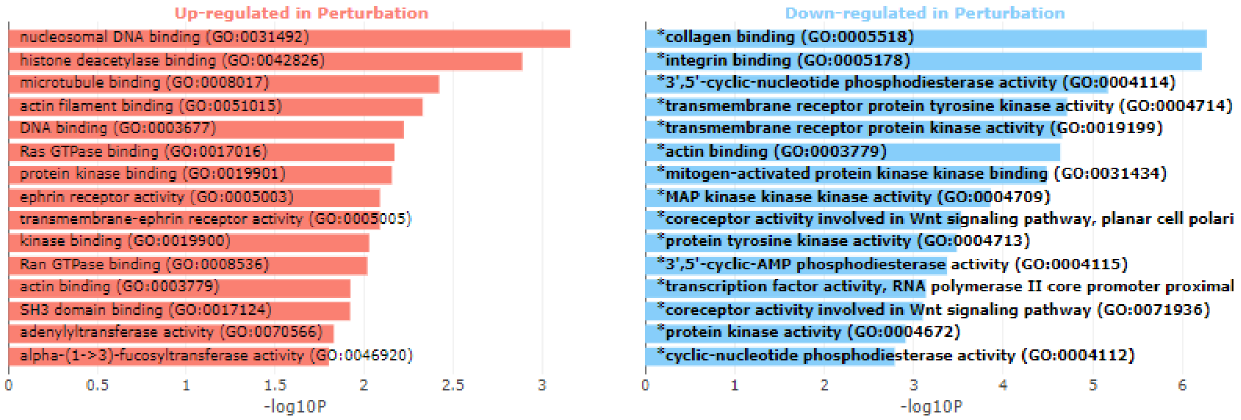

The figure contains interactive bar charts displaying the results of the Gene Ontology enrichment analysis generated using Enrichr. The x-axis indicates the -log10(*P*-value) for each term. Significant terms are highlighted in bold.
