# Supplementary material for: Metabolic changes preceding bladder cancer occurrence among Korean men: a nested case-control study from the KCPS-II cohort
Source: Cancer Metab. 2023 Dec 5;11:23. doi: 10.1186/s40170-023-00324-0 (PMC10696702; doi:10.1186/s40170-023-00324-0)
Supplement: Supplementary file 2 — Additional file 2. Supplementary Table S1. SNPs used to construct GRS. [file 40170_2023_324_MOESM2_ESM.docx]

**Table S1. SNPs used to construct GRS**

| **SNP** | **Position** | **Gene** | **SNP** | **Position** | **Gene** |
| --- | --- | --- | --- | --- | --- |
| rs146300746 | chr1:10744890 | CASZ1 | rs2267889 | chr7:108377193 | NRCAM |
| rs1561597 | chr1:82988544 | LINC01362 | rs144615174 | chr7:154013539 | DPP6 |
| rs11437221 | chr1:96576201-96576215 | - | rs6470670 | chr8:128901202 | - |
| rs72721996 | chr1:96809637 | PTBP2 | rs75860110 | chr8:1444560 | DLGAP2 |
| rs74225595 | chr1:11578610 | - | rs138888197 | chr8:64779617-64779625 | CYP7B1 |
| rs1540925 | chr1:186025891 | HMCN1 | rs10088569 | chr8:1407947 | DLGAP2 |
| rs200099269 | chr1:85443410 | DDAH1 | rs117726046 | chr8:43399376 | - |
| rs146716993 | chr2:103408248 | - | rs145168239 | chr9:21315190 | LOC107987053 |
| rs6542613 | chr2:4596219 | - | rs7914359 | chr10:1603852 | ADARB2 |
| rs35204237 | chr2:154702614 | KCNJ3 | rs74585395 | chr10:1567778 | ADARB2 |
| rs117070880 | chr2:195976841 | DNAH7 | rs140507858 | chr10:71878620 | - |
| rs55794119 | chr2:34547699 | - | rs76034296 | chr10:71996905 | CHST3 |
| rs76342099 | chr2:206720736 | - | rs144683342 | chr10:3482293 | LINC02669 |
| rs150815912 | chr3:152123029 | - | rs74318518 | chr11:110971294 | - |
| rs9819131 | chr3:134946091 | EPHB1 | rs143900019 | chr11:15420653 | - |
| rs28750517 | chr3:145279920 | - | rs182746841 | chr11:16341268 | SOX6 |
| rs117195185 | chr3:41739082 | ULK4 | rs145542969 | chr11:78735839-78735842 | TENM4 |
| rs150692716 | chr3:36027984-36028008 | - | rs149616675 | chr11:30029569 | LOC107984321 |
| rs12632137 | chr3:72236544 | - | rs151007360 | chr13:61690905 | - |
| rs77230313 | chr3:151173607 | MED12L | rs9552477 | chr13:21731852 | - |
| rs61382574 | chr3:196039134 | TFRC | rs28608483 | chr14:62908341 | KCNH5 |
| rs146380721 | chr3:36280879 | - | rs117802386 | chr14:47464251 | MDGA2 |
| rs139606103 | chr3:23132367 | - | rs148637203 | chr14:92807473 | GOLGA5 |
| rs184072744 | chr3:196813427 | PAK2 | rs876525 | chr14:65191477 | - |
| rs35797617 | chr4:47090838-47090843 | GABRB1 | rs10467941 | chr15:69659567 | PCAT29 |
| rs148403929 | chr4:136560692 | - | rs151104101 | chr15:55473971 | DNAAF4 |
| rs17865157 | chr4:117486396 | LINC01378 | rs62049086 | chr16:22914936 | HS3ST2 |
| rs76802842 | chr4:163927901 | MARCHF1 | rs4564573 | chr16:25969550 | HS3ST4 |
| rs138655958 | chr4:166744488 | SPOCK3 | rs138426339 | chr17:78838829 | USP36 |
| rs190509954 | chr5:76846518 | - | rs12953114 | chr17:13214602 | - |
| rs74389478 | chr5:54223209 | ARL15 | rs9915078 | chr17:35116196 | RAD51D |
| rs79138763 | chr5:122576834 | - | rs7222961 | chr17:8693590 | LOC105371525 |
| rs466506 | chr5:115701027 | - | rs73990429 | chr17:55521571 | - |
| rs77330218 | chr5:17200379 | BASP1-AS1 | rs12951350 | chr17:35084975 | RFFL |
| rs117560137 | chr5:84513606 | EDIL3-DT | rs580735 | chr18:26681571 | LOC102725227 |
| rs499225 | chr5:38882145 | OSMR | rs115700232 | chr18:45310686 | SLC14A2 |
| rs77417497 | chr5:179443952 | - | rs73045226 | chr19:41255008 | AXL |
| rs58259903 | chr5:11560444 | CTNND2 | rs148150089 | chr20:23066670 | - |
| rs74325657 | chr6:21016777 | CDKAL1 | rs117202500 | chr20:54278757 | - |
| rs117757835 | chr6:24239298 | DCDC2 | rs139048822 | chr20:8109325-8109328 | - |
| rs201651729 | chr6:12313906 | - | rs77137500 | chr20:10814537 | - |
| rs142341710 | chr6:53154359 | GCM1 | rs389468 | chr21:14222144 | RBM11 |
| rs7766508 | chr6:161372287 | PRKN | rs560990092 | chr21:26193552-26193570 | - |
| rs34386486 | chr6:106531652-106531664 | CRYBG1 | rs5750424 | chr22:37342910 | ELFN2 |
| rs138690633 | chr7:89198816-89198820 | ZNF804B | rs116999260 | chr7:6130630 | USP42 |
| rs34271250 | chr7:156341361 |  | rs4720240 | chr7:37167124 | ELMO1 |
