# Supplementary material for: Metabolic changes preceding bladder cancer occurrence among Korean men: a nested case-control study from the KCPS-II cohort
Source: Cancer Metab. 2023 Dec 5;11:23. doi: 10.1186/s40170-023-00324-0 (PMC10696702; doi:10.1186/s40170-023-00324-0)
Supplement: Supplementary file 3 — Additional file 3. Supplementary Table S2. Baseline clinical and biochemical characteristics of subjects (all). [file 40170_2023_324_MOESM3_ESM.docx]

**Table S2. Baseline clinical and biochemical characteristics of subjects (all)**

|  | **All** | | | | | | |
| --- | --- | --- | --- | --- | --- | --- | --- |
|  | **Total (*n*=132)** | | | | | | ***p*** |
|  | **Control (*n*=66)** | | | **Bladder cancer occurrence (*n*=66)** | | |  |
| Age (year) | 51.12 | | ±1.02 | 52.50 | | ±1.08 | 0.252*^†^* |
| Current smoker *n,* (%) | 27 | (40.9) | | 32 | (48.5) | | 0.119 |
| Body mass index (kg/m^2^) | 24.28 | | ±0.31 | 24.81 | | ±0.32 | 0.243 |
| Waist circumference (cm) | 85.71 | | ±0.86 | 87.21 | | ±0.82 | 0.161*^†^* |
| Systolic blood pressure (mmHg) | 124.70 | | ±1.81 | 122.04 | | ±1.85 | 0.292*^∮^* |
| Diastolic blood pressure (mmHg) | 79.86 | | ±1.26 | 75.85 | | ±1.15 | 0.062*^†^* |
| Glucose (mg/dL) | 96.39 | | ±2.68 | 97.40 | | ±3.00 | 0.838*^†^* |
| White blood cell (10^3^/μL ) | 6.01 | | ±0.18 | 6.44 | | ±0.18 | 0.097*^†^* |
| Albumin (g/dL) | 4.56 | | ±0.04 | 4.54 | | ±0.03 | 0.696 |
| Total cholesterol (mg/dL) | 190.01 | | ±4.56 | 198.47 | | ±4.27 | 0.175 |
| Triglyceride (mg/dL) | 165.30 | | ±11.27 | 158.71 | | ±9.68 | 0.706*^∮^* |
| HDL-cholesterol (mg/dL) | 47.89 | | ±1.14 | 47.91 | | ±0.89 | 0.856*^∮^* |
| LDL-cholesterol (mg/dL) | 113.67 | | ±4.22 | 120.93 | | ±4.10 | 0.225 |
| AST (IU/L) | 31.52 | | ±3.36 | 28.33 | | ±3.44 | 0.458*^†^* |
| ALT (IU/L) | 36.31 | | ±5.43 | 30.84 | | ±2.48 | 0.974*^†^* |
| GGT (IU/L) | 77.65 | | ±13.63 | 52.45 | | ±4.93 | 0.303*^†^* |
| Bilirubin (mg/dL) | 0.94 | | ±0.05 | 0.91 | | ±0.04 | 0.942*^†^* |
| Uric acid (mg/dL) | 5.94 | | ±0.18 | 6.06 | | ±0.15 | 0.615 |
| Blood urea nitrogen (mg/dL) | 15.81 | | ±0.99 | 15.01 | | ±0.51 | 0.885*^†^* |
| Creatinine (mg/dL) | 1.20 | | ±0.10 | 1.06 | | ±0.02 | 0.357*^†^* |

Mean ± standard error (SE). Comparisons were conducted between the two groups (control *vs.* bladder cancer occurrence). Continuous variables were tested by an independent t-test, and variables marked with *∮* were tested by logarithmic transformation. Continuous variables with a nonnormal distribution even after logarithmic transformation were tested by a Mann-Whitney U test, and *p*-values are marked with *†*. Smoking status was tested by a Chi-squared test. AST: aspartate aminotransferase. ALT: alanine aminotransferase. GGT: γ-glutamyltransferase. HDL: high-density lipoprotein. LDL: low-density lipoprotein.
