# Supplementary material for: Metabolic changes preceding bladder cancer occurrence among Korean men: a nested case-control study from the KCPS-II cohort
Source: Cancer Metab. 2023 Dec 5;11:23. doi: 10.1186/s40170-023-00324-0 (PMC10696702; doi:10.1186/s40170-023-00324-0)
Supplement: Supplementary file 4 — Additional file 4. Supplementary Table S3. Baseline clinical and biochemical characteristics of BLCA subjects. [file 40170_2023_324_MOESM4_ESM.docx]

**Table S3. Baseline clinical and biochemical characteristics of BLCA subjects**

|  | **BLCA subjects** | | | | | | |
| --- | --- | --- | --- | --- | --- | --- | --- |
|  | **Total (*n*=64)** | | | | | | ***p*** |
|  | **GRS-low (*n*=32)** | | | **GRS-high (*n*=32)** | | |  |
| Genetic risk score | 39.51 | | ±0.67 | 53.59 | | ±1.11 | **<0.001** |
| Age (year) | 53.18 | | ±1.65 | 52.9 | | ±1.33 | 0.906 |
| Current smoker *n,* (%) | 15 | (46.9) | | 15 | (46.9) | | 0.914 |
| Body mass index (kg/m^2^) | 24.69 | | ±0.50 | 25.02 | | ±0.42 | 0.546 |
| Waist circumference (cm) | 86.94 | | ±1.22 | 87.37 | | ±1.16 | 0.757 |
| Systolic blood pressure (mmHg) | 119.30 | | ±2.26 | 126.25 | | ±2.83 | 0.059 |
| Diastolic blood pressure (mmHg) | 73.87 | | ±1.63 | 78.54 | | ±1.54 | **0.046** |
| Glucose (mg/dL) | 98.72 | | ±4.60 | 97.03 | | ±4.14 | 0.727*^†^* |
| White blood cell (10^3^/μL ) | 6.64 | | ±0.27 | 6.27 | | ±0.25 | 0.350*^∮^* |
| Albumin (g/dL) | 4.54 | | ±0.05 | 4.53 | | ±0.05 | 0.974 |
| Total cholesterol (mg/dL) | 204.01 | | ±5.69 | 191.72 | | ±6.46 | 0.160 |
| Triglyceride (mg/dL) | 147.90 | | ±13.31 | 167.59 | | ±14.18 | 0.211*^∮^* |
| HDL-cholesterol (mg/dL) | 48.02 | | ±1.27 | 48.04 | | ±1.28 | 0.968 |
| LDL-cholesterol (mg/dL) | 126.61 | | ±5.32 | 113.49 | | ±6.18 | 0.112 |
| AST (IU/L) | 25.61 | | ±1.33 | 31.63 | | ±7.09 | 0.615*^†^* |
| ALT (IU/L) | 28.77 | | ±2.54 | 32.87 | | ±4.45 | 0.896*^†^* |
| GGT (IU/L) | 46.80 | | ±5.51 | 59.32 | | ±8.44 | 0.464*^†^* |
| Bilirubin (mg/dL) | 0.96 | | ±0.05 | 0.86 | | ±0.07 | 0.261 |
| Uric acid (mg/dL) | 5.84 | | ±0.22 | 6.29 | | ±0.22 | 0.153 |
| Blood urea nitrogen (mg/dL) | 15.54 | | ±0.70 | 14.38 | | ±0.80 | 0.299 |
| Creatinine (mg/dL) | 1.04 | | ±0.03 | 1.07 | | ±0.03 | 0.617 |

Mean ± standard error (SE). Comparisons were conducted between the two groups (GRS-high *vs.* GRS-low). BLCA was divided into two groups by median of GRS. Continuous variables were tested by an independent t-test, and variables marked with *∮* were tested by logarithmic transformation. Even after logarithmic transformation, continuous variables with a nonnormal distribution were tested by a Mann-Whitney U test, and p-values were marked with *†*. Smoking status was tested by a Chi-squared test. AST: aspartate aminotransferase. ALT: alanine aminotransferase. GGT: γ-glutamyltransferase. HDL: high-density lipoprotein. LDL: low-density lipoprotein.
