# Supplementary material for: Metabolic changes preceding bladder cancer occurrence among Korean men: a nested case-control study from the KCPS-II cohort
Source: Cancer Metab. 2023 Dec 5;11:23. doi: 10.1186/s40170-023-00324-0 (PMC10696702; doi:10.1186/s40170-023-00324-0)
Supplement: Supplementary file 5 — Additional file 5. Supplementary Table S4. LC/MS method validation. [file 40170_2023_324_MOESM5_ESM.docx]

**Table S4. LC/MS method validation**

| **Peak intensity of L-leucine-^13^C** | | | | |
| --- | --- | --- | --- | --- |
|  | **1day**  **QC1** | **2day**  **QC1** | **1day**  **QC2** | **2day**  **QC2** |
| 1 | 1252201003 | 1260818734 | 1321600649 | 1244057565 |
| 2 | 1282721293 | 1288032663 | 1293712246 | 1214104940 |
| 3 | 1306240161 | 1231806527 | 1278063679 | 1263347969 |
| 4 | 1267430569 | 1286637263 | 1263453172 | 1310845726 |
| 5 | 1324765721 | 1240075487 | 1240930542 | 1346737267 |
| 6 | 1260584789 | 1301506726 | 1268602111 | 1267633832 |
| 7 | 1287669688 | 1242466604 | 1309386549 | 1223742140 |
| **Intra-assay RSD (%)** |  |  |  |  |
|  | 2.012 | 2.184 | 2.178 | 3.742 |
| **Inter-assay RSD (%)** |  |  |  |  |
|  | 2.154 | | 2.996 | |

RSD, relative standard deviation. L-leucine-^13^C was obtained in ESI-positive ion mode. Intra-assay measured the variance of QC sample replicates within a day. Inter-assay measured the variance between sample replicates on a different day.
