# Supplementary material for: Metabolic changes preceding bladder cancer occurrence among Korean men: a nested case-control study from the KCPS-II cohort
Source: Cancer Metab. 2023 Dec 5;11:23. doi: 10.1186/s40170-023-00324-0 (PMC10696702; doi:10.1186/s40170-023-00324-0)
Supplement: Supplementary file 6 — Additional file 6. Supplementary Table S5. Metabolite set analysis for super-class associated with bladder cancer risk in all subjects. [file 40170_2023_324_MOESM6_ESM.docx]

**Table S5. Metabolite set analysis for super-class associated with bladder cancer risk in all subjects**

| **Super-class** | **Contributing metabolites** | ***p*** | **FDR** |
| --- | --- | --- | --- |
| Organic acids | Betaine Phenylalanine Alanine Threonine Histidine Lysine Aspartic acid Taurine Arginine Succinylacetone Tryptophan Suberic acid | 5.72E-26 | **5.15E-25** |
| Polyketides | Cinnamic acid | 1.53E-22 | **6.89E-22** |
| Benzenoids | Toluene Benzaldehyde | 6.12E-20 | **1.84E-19** |
| Organic oxygen compounds | 4-Hydroxybenzaldehyde Acetophenone | 1.26E-18 | **2.84E-18** |
| Organoheterocyclic compounds | Biotin Indoleacrylic acid Indole Indoleacetaldehyde | 1.82E-07 | **3.27E-07** |
| Fatty Acyls | 3-Hydroxysebacic acid Glutaric acid Palmitoylethanolamide Oleoylethanolamine Heptanoylcarnitine | 0.000113 | **0.000169** |
| Glycerophospholipids | LysoPC(20:5/0:0)  LysoPC(P-16:0/0:0) LysoPE(20:0/0:0) | 0.0716 | 0.0920 |
| Sphingolipids | Sphinganine Phytosphingosine | 0.114 | 0.128 |
| Lipids and lipid-like molecules | 6-Hydroxypentadecanedioic acid | 0.182 | 0.182 |

Metabolites with variable importance in the projection (VIP) values ​​> 1.0 in both sets were used for metabolite set analysis. *p*-values obtained by the metabolite set analysis were adjusted by false discovery rate (FDR) method to control a multiple testing error.
