# Supplementary material for: Metabolic changes preceding bladder cancer occurrence among Korean men: a nested case-control study from the KCPS-II cohort
Source: Cancer Metab. 2023 Dec 5;11:23. doi: 10.1186/s40170-023-00324-0 (PMC10696702; doi:10.1186/s40170-023-00324-0)
Supplement: Supplementary file 7 — Additional file 7. Supplementary Table S6. Logistic regression analysis to identify smoking-related metabolites in BLCA. [file 40170_2023_324_MOESM7_ESM.docx]

**Table S6. Logistic regression analysis to identify smoking-related metabolites in BLCA**

|  | | **Exp(B)** | **S.E.** | **Wald** | ***p*** |
| --- | --- | --- | --- | --- | --- |
| Current  smoking  status | Lysine | 0.997 | 0.003 | 0.837 | 0.360 |
|  | Indoleacrylic acid | 1.002 | 0.010 | 0.032 | 0.857 |
|  | Tryptophan | 0.999 | 0.010 | 0.004 | 0.951 |
|  | Indole | 0.970 | 0.021 | 1.944 | 0.163 |
|  | Indoleacetaldehyde | 0.976 | 0.241 | 0.010 | 0.919 |
|  | Toluene | 1.225 | 0.123 | 2.720 | 0.099 |
|  | Benzaldehyde | 1.011 | 0.008 | 1.831 | 0.176 |
|  | Betaine | 0.999 | 0.001 | 1.500 | 0.221 |
|  | **Threonine** | 1.041 | 0.018 | 5.114 | **0.024** |
|  | **Acetophenone** | 0.782 | 0.110 | 4.997 | **0.025** |
|  | 4-Hydroxybenzaldehyde | 1.012 | 0.010 | 1.567 | 0.211 |
|  | Glutaric acid | 0.249 | 1.342 | 1.074 | 0.300 |
|  | Aspartic acid | 1.050 | 0.061 | 0.650 | 0.420 |
|  | Cinnamic acid | 0.993 | 0.004 | 2.876 | 0.090 |
|  | Histidine | 0.998 | 0.004 | 0.193 | 0.661 |
|  | Succinylacetone | 1.070 | 0.113 | 0.355 | 0.551 |
|  | Phenylalanine | 1.007 | 0.004 | 2.948 | 0.086 |
|  | Suberic acid | 1.010 | 0.033 | 0.095 | 0.758 |
|  | Arginine | 0.997 | 0.001 | 3.290 | 0.070 |
|  | 3-Hydroxysebacic acid | 1.107 | 0.094 | 1.160 | 0.281 |
|  | Biotin | 0.995 | 0.010 | 0.287 | 0.592 |
|  | Palmitoylethanolamide | 0.984 | 0.017 | 0.883 | 0.347 |
|  | Oleoylethanolamide | 1.011 | 0.026 | 0.164 | 0.685 |
