# Supplementary material for: Metabolic changes preceding bladder cancer occurrence among Korean men: a nested case-control study from the KCPS-II cohort
Source: Cancer Metab. 2023 Dec 5;11:23. doi: 10.1186/s40170-023-00324-0 (PMC10696702; doi:10.1186/s40170-023-00324-0)
Supplement: Supplementary file 8 — Additional file 8. Supplementary Table S7. Gene Ontology Enrichment Analysis Results of our data using FUMA. [file 40170_2023_324_MOESM8_ESM.docx]

**Table S7. Gene Ontology Enrichment Analysis Results of our data using FUMA**

[**Gene Ontology Biological Processes**](https://fuma.ctglab.nl/gene2func/95220#GO_bpPanel)

| **GeneSet** | ***P*** | **Adjusted *P*** | **Genes** |
| --- | --- | --- | --- |
| REGULATION_OF_PEPTIDYL_SERINE_PHOSPHORYLATION_OF_STAT_PROTEIN | 1.15e-15 | 8.45e-12 | IFNA21, IFNA16, IFNA17, IFNA14, IFNA5, IFNA6, IFNA2 |
| [SERINE_PHOSPHORYLATION_OF_STAT_PROTEIN](http://www.gsea-msigdb.org/gsea/msigdb/cards/GO_SERINE_PHOSPHORYLATION_OF_STAT_PROTEIN) | 4.15e-15 | 1.52e-11 | IFNA21, IFNA16, IFNA17, IFNA14, IFNA5, IFNA6, IFNA2 |
| [NATURAL_KILLER_CELL_ACTIVATION_INVOLVED_IN_IMMUNE_RESPONSE](http://www.gsea-msigdb.org/gsea/msigdb/cards/GO_NATURAL_KILLER_CELL_ACTIVATION_INVOLVED_IN_IMMUNE_RESPONSE) | 1.22e-14 | 3.00e-11 | IFNA21, IFNA16, IFNA17, IFNA14, IFNA5, IFNA6, IFNA2 |
| [NATURAL_KILLER_CELL_ACTIVATION](http://www.gsea-msigdb.org/gsea/msigdb/cards/GO_NATURAL_KILLER_CELL_ACTIVATION) | 1.35e-13 | 2.49e-10 | AXL, IFNA21, IFNA16, IFNA17, IFNA14, IFNA5, IFNA6, IFNA2 |
| [RESPONSE_TO_DSRNA](http://www.gsea-msigdb.org/gsea/msigdb/cards/GO_RESPONSE_TO_DSRNA) | 7.03e-13 | 8.85e-10 | IFNA21, IFNA16, IFNA17, IFNA14, IFNA5, IFNA6, IFNA2 |
| [T_CELL_ACTIVATION_INVOLVED_IN_IMMUNE_RESPONSE](http://www.gsea-msigdb.org/gsea/msigdb/cards/GO_T_CELL_ACTIVATION_INVOLVED_IN_IMMUNE_RESPONSE) | 7.23e-13 | 8.85e-10 | F2RL1, IFNA21, IFNA16, IFNA17, IFNA14, IFNA5, IFNA6, IFNA2 |
| [POSITIVE_REGULATION_OF_PEPTIDYL_SERINE_PHOSPHORYLATION](http://www.gsea-msigdb.org/gsea/msigdb/cards/GO_POSITIVE_REGULATION_OF_PEPTIDYL_SERINE_PHOSPHORYLATION) | 1.18e-12 | 1.16e-9 | TGFB1, IFNA21, IFNA16, IFNA17, IFNA14, IFNA5, IFNA6, IFNA2 |
| [LYMPHOCYTE_ACTIVATION_INVOLVED_IN_IMMUNE_RESPONSE](http://www.gsea-msigdb.org/gsea/msigdb/cards/GO_LYMPHOCYTE_ACTIVATION_INVOLVED_IN_IMMUNE_RESPONSE) | 1.26e-12 | 1.16e-9 | TGFB1, F2RL1, IFNA21, IFNA16, IFNA17, IFNA14, IFNA5, IFNA6, IFNA2 |
| [REGULATION_OF_PEPTIDYL_SERINE_PHOSPHORYLATION](http://www.gsea-msigdb.org/gsea/msigdb/cards/GO_REGULATION_OF_PEPTIDYL_SERINE_PHOSPHORYLATION) | 9.84e-12 | 8.04e-9 | TGFB1, IFNA21, IFNA16, IFNA17, IFNA14, IFNA5, IFNA6, IFNA2 |
| [RESPONSE_TO_TYPE_I_INTERFERON](http://www.gsea-msigdb.org/gsea/msigdb/cards/GO_RESPONSE_TO_TYPE_I_INTERFERON) | 2.38e-11 | 1.75e-8 | IFNA21, IFNA16, IFNA17, IFNA14, IFNA5, IFNA6, IFNA2 |
| [LEUKOCYTE_DIFFERENTIATION](http://www.gsea-msigdb.org/gsea/msigdb/cards/GO_LEUKOCYTE_DIFFERENTIATION) | 2.84e-11 | 1.90e-8 | AXL, TGFB1, F2RL1, EIF2AK1, IFNA21, IFNA16, IFNA17, IFNA14, IFNA5, IFNA6, IFNA2 |
| [B_CELL_PROLIFERATION](http://www.gsea-msigdb.org/gsea/msigdb/cards/GO_B_CELL_PROLIFERATION) | 4.17e-11 | 2.55e-8 | IFNA21, IFNA16, IFNA17, IFNA14, IFNA5, IFNA6, IFNA2 |
| [LEUKOCYTE_PROLIFERATION](http://www.gsea-msigdb.org/gsea/msigdb/cards/GO_LEUKOCYTE_PROLIFERATION) | 9.42e-11 | 5.32e-8 | TGFB1, F2RL1, IFNA21, IFNA16, IFNA17, IFNA14, IFNA5, IFNA6, IFNA2 |
| [RESPONSE_TO_VIRUS](http://www.gsea-msigdb.org/gsea/msigdb/cards/GO_RESPONSE_TO_VIRUS) | 2.77e-10 | 1.46e-7 | HNRNPUL1, F2RL1, IFNA21, IFNA16, IFNA17, IFNA14, IFNA5, IFNA6, IFNA2 |
| [B_CELL_DIFFERENTIATION](http://www.gsea-msigdb.org/gsea/msigdb/cards/GO_B_CELL_DIFFERENTIATION) | 3.60e-10 | 1.77e-7 | IFNA21, IFNA16, IFNA17, IFNA14, IFNA5, IFNA6, IFNA2 |
| [**LYMPHOCYTE_ACTIVATION**](http://www.gsea-msigdb.org/gsea/msigdb/cards/GO_LYMPHOCYTE_ACTIVATION) | 4.02e-10 | 1.85e-7 | **AXL**, TGFB1, **EPHB1**, F2RL1, IFNA21, IFNA16, IFNA17, IFNA14, IFNA5, IFNA6, IFNA2 |
| [REGULATION_OF_RECEPTOR_SIGNALING_PATHWAY_VIA_STAT](http://www.gsea-msigdb.org/gsea/msigdb/cards/GO_REGULATION_OF_RECEPTOR_SIGNALING_PATHWAY_VIA_STAT) | 4.74e-10 | 2.01e-7 | IFNA21, IFNA16, IFNA17, IFNA14, IFNA5, IFNA6, IFNA2 |
| [**COAGULATION**](http://www.gsea-msigdb.org/gsea/msigdb/cards/GO_COAGULATION) | 4.91e-10 | 2.01e-7 | **AXL**, F2RL1, IFNA21, IFNA16, IFNA17, IFNA14, IFNA5, IFNA6, IFNA2 |
| [**LYMPHOCYTE_DIFFERENTIATION**](http://www.gsea-msigdb.org/gsea/msigdb/cards/GO_LYMPHOCYTE_DIFFERENTIATION) | 5.75e-10 | 2.22e-7 | **AXL**, TGFB1, IFNA21, IFNA16, IFNA17, IFNA14, IFNA5, IFNA6, IFNA2 |
| [DEFENSE_RESPONSE_TO_VIRUS](http://www.gsea-msigdb.org/gsea/msigdb/cards/GO_DEFENSE_RESPONSE_TO_VIRUS) | 7.51e-10 | 2.76e-7 | F2RL1, IFNA21, IFNA16, IFNA17, IFNA14, IFNA5, IFNA6, IFNA2 |
| [B_CELL_ACTIVATION](http://www.gsea-msigdb.org/gsea/msigdb/cards/GO_B_CELL_ACTIVATION) | 8.89e-10 | 3.11e-7 | TGFB1, IFNA21, IFNA16, IFNA17, IFNA14, IFNA5, IFNA6, IFNA2 |
| [RECEPTOR_SIGNALING_PATHWAY_VIA_STAT](http://www.gsea-msigdb.org/gsea/msigdb/cards/GO_RECEPTOR_SIGNALING_PATHWAY_VIA_STAT) | 1.29e-9 | 4.30e-7 | IFNA21, IFNA16, IFNA17, IFNA14, IFNA5, IFNA6, IFNA2 |
| [**WOUND_HEALING**](http://www.gsea-msigdb.org/gsea/msigdb/cards/GO_WOUND_HEALING) | 1.39e-9 | 4.45e-7 | **AXL**, TGFB1, F2RL1, IFNA21, IFNA16, IFNA17, IFNA14, IFNA5, IFNA6, IFNA2 |
| [T_CELL_ACTIVATION](http://www.gsea-msigdb.org/gsea/msigdb/cards/GO_T_CELL_ACTIVATION) | 5.69e-9 | 1.74e-6 | TGFB1, F2RL1, IFNA21, IFNA16, IFNA17, IFNA14, IFNA5, IFNA6, IFNA2 |
| [PEPTIDYL_SERINE_MODIFICATION](http://www.gsea-msigdb.org/gsea/msigdb/cards/GO_PEPTIDYL_SERINE_MODIFICATION) | 6.47e-9 | 1.90e-6 | TGFB1, IFNA21, IFNA16, IFNA17, IFNA14, IFNA5, IFNA6, IFNA2 |
| [**RESPONSE_TO_WOUNDING**](http://www.gsea-msigdb.org/gsea/msigdb/cards/GO_RESPONSE_TO_WOUNDING) | 8.46e-9 | 2.39e-6 | **AXL**, TGFB1, F2RL1, IFNA21, IFNA16, IFNA17, IFNA14, IFNA5, IFNA6, IFNA2 |
| [DEFENSE_RESPONSE_TO_OTHER_ORGANISM](http://www.gsea-msigdb.org/gsea/msigdb/cards/GO_DEFENSE_RESPONSE_TO_OTHER_ORGANISM) | 1.06e-8 | 2.89e-6 | TGFB1, F2RL1, IFNA21, IFNA16, IFNA17, IFNA14, IFNA5, IFNA6, IFNA2 |
| [**REGULATION_OF_BODY_FLUID_LEVELS**](http://www.gsea-msigdb.org/gsea/msigdb/cards/GO_REGULATION_OF_BODY_FLUID_LEVELS) | 1.39e-8 | 3.64e-6 | **AXL**, F2RL1, IFNA21, IFNA16, IFNA17, IFNA14, IFNA5, IFNA6, IFNA2 |
| [**RESPONSE_TO_BIOTIC_STIMULUS**](http://www.gsea-msigdb.org/gsea/msigdb/cards/GO_RESPONSE_TO_BIOTIC_STIMULUS) | 2.09e-8 | 5.30e-6 | **AXL**, HNRNPUL1, TGFB1, F2RL1, IFNA21, IFNA16, IFNA17, IFNA14, IFNA5, IFNA6, IFNA2 |
| [**IMMUNE_SYSTEM_DEVELOPMENT**](http://www.gsea-msigdb.org/gsea/msigdb/cards/GO_IMMUNE_SYSTEM_DEVELOPMENT) | 2.41e-8 | 5.91e-6 | **AXL**, TGFB1, F2RL1, EIF2AK1, IFNA21, IFNA16, IFNA17, IFNA14, IFNA5, IFNA6, IFNA2 |
| [ADAPTIVE_IMMUNE_RESPONSE](http://www.gsea-msigdb.org/gsea/msigdb/cards/GO_ADAPTIVE_IMMUNE_RESPONSE) | 3.10e-8 | 6.98e-6 | TGFB1, IFNA21, IFNA16, IFNA17, IFNA14, IFNA5, IFNA6, IFNA2 |
| [HUMORAL_IMMUNE_RESPONSE](http://www.gsea-msigdb.org/gsea/msigdb/cards/GO_HUMORAL_IMMUNE_RESPONSE) | 3.11e-8 | 6.98e-6 | IFNA21, IFNA16, IFNA17, IFNA14, IFNA5, IFNA6, IFNA2 |
| [**CYTOKINE_MEDIATED_SIGNALING_PATHWAY**](http://www.gsea-msigdb.org/gsea/msigdb/cards/GO_CYTOKINE_MEDIATED_SIGNALING_PATHWAY) | 3.14e-8 | 6.98e-6 | **AXL**, TGFB1, F2RL1, IFNA21, IFNA16, IFNA17, IFNA14, IFNA5, IFNA6, IFNA2 |
| [**INNATE_IMMUNE_RESPONSE**](http://www.gsea-msigdb.org/gsea/msigdb/cards/GO_INNATE_IMMUNE_RESPONSE) | 1.21e-7 | 2.62e-5 | **AXL**, TGFB1, F2RL1, IFNA21, IFNA16, IFNA17, IFNA14, IFNA5, IFNA6, IFNA2 |
| [CELL_ACTIVATION_INVOLVED_IN_IMMUNE_RESPONSE](http://www.gsea-msigdb.org/gsea/msigdb/cards/GO_CELL_ACTIVATION_INVOLVED_IN_IMMUNE_RESPONSE) | 2.61e-7 | 5.49e-5 | TGFB1, F2RL1, IFNA21, IFNA16, IFNA17, IFNA14, IFNA5, IFNA6, IFNA2 |
| [**CELL_ACTIVATION**](http://www.gsea-msigdb.org/gsea/msigdb/cards/GO_CELL_ACTIVATION) | 7.21e-7 | 1.47e-4 | **AXL**, TGFB1, **EPHB1**, F2RL1, IFNA21, IFNA16, IFNA17, IFNA14, IFNA5, IFNA6, IFNA2 |
| [REGULATION_OF_SIGNALING_RECEPTOR_ACTIVITY](http://www.gsea-msigdb.org/gsea/msigdb/cards/GO_REGULATION_OF_SIGNALING_RECEPTOR_ACTIVITY) | 9.39e-7 | 1.87e-4 | TGFB1, IFNA21, IFNA16, IFNA17, IFNA14, IFNA5, IFNA6, IFNA2 |
| [**IMMUNE_EFFECTOR_PROCESS**](http://www.gsea-msigdb.org/gsea/msigdb/cards/GO_IMMUNE_EFFECTOR_PROCESS) | 1.50e-6 | 2.90e-4 | TGFB1, F2RL1, **ELMO1**, IFNA21, IFNA16, IFNA17, IFNA14, IFNA5, IFNA6, IFNA2 |
| [**RESPONSE_TO_CYTOKINE**](http://www.gsea-msigdb.org/gsea/msigdb/cards/GO_RESPONSE_TO_CYTOKINE) | 1.63e-6 | 3.07e-4 | **AXL**, TGFB1, F2RL1, IFNA21, IFNA16, IFNA17, IFNA14, IFNA5, IFNA6, IFNA2 |
| [**PEPTIDYL_AMINO_ACID_MODIFICATION**](http://www.gsea-msigdb.org/gsea/msigdb/cards/GO_PEPTIDYL_AMINO_ACID_MODIFICATION) | 2.68e-6 | 4.93e-4 | **AXL**, TGFB1, **EPHB1**, IFNA21, IFNA16, IFNA17, IFNA14, IFNA5, IFNA6, IFNA2 |
| [**DEFENSE_RESPONSE**](http://www.gsea-msigdb.org/gsea/msigdb/cards/GO_DEFENSE_RESPONSE) | 3.26e-6 | 5.84e-4 | **AXL**, TGFB1, F2RL1, EIF2AK1, IFNA21, IFNA16, IFNA17, IFNA14, IFNA5, IFNA6, IFNA2 |
| [**PROTEIN_PHOSPHORYLATION**](http://www.gsea-msigdb.org/gsea/msigdb/cards/GO_PROTEIN_PHOSPHORYLATION) | 3.48e-6 | 6.09e-4 | **AXL**, TGFB1, **EPHB1**, F2RL1, EIF2AK1, IFNA21, IFNA16, IFNA17, IFNA14, IFNA5, IFNA6, IFNA2 |
| [**REGULATION_OF_PHOSPHORUS_METABOLIC_PROCESS**](http://www.gsea-msigdb.org/gsea/msigdb/cards/GO_REGULATION_OF_PHOSPHORUS_METABOLIC_PROCESS) | 5.35e-6 | 9.15e-4 | TGFB1, **EPHB1**, F2RL1, EIF2AK1, IFNA21, IFNA16, IFNA17, IFNA14, IFNA5, IFNA6, IFNA2 |
| [POSITIVE_REGULATION_OF_PHOSPHORUS_METABOLIC_PROCESS](http://www.gsea-msigdb.org/gsea/msigdb/cards/GO_POSITIVE_REGULATION_OF_PHOSPHORUS_METABOLIC_PROCESS) | 7.80e-6 | 1.30e-3 | TGFB1, F2RL1, IFNA21, IFNA16, IFNA17, IFNA14, IFNA5, IFNA6, IFNA2 |
| [**REGULATION_OF_PROTEIN_MODIFICATION_PROCESS**](http://www.gsea-msigdb.org/gsea/msigdb/cards/GO_REGULATION_OF_PROTEIN_MODIFICATION_PROCESS) | 1.24e-5 | 2.02e-3 | TGFB1, **EPHB1**, F2RL1, EIF2AK1, IFNA21, IFNA16, IFNA17, IFNA14, IFNA5, IFNA6, IFNA2 |
| [POSITIVE_REGULATION_OF_PROTEIN_MODIFICATION_PROCESS](http://www.gsea-msigdb.org/gsea/msigdb/cards/GO_POSITIVE_REGULATION_OF_PROTEIN_MODIFICATION_PROCESS) | 2.08e-5 | 3.31e-3 | TGFB1, F2RL1, IFNA21, IFNA16, IFNA17, IFNA14, IFNA5, IFNA6, IFNA2 |
| [**PHAGOCYTOSIS**](http://www.gsea-msigdb.org/gsea/msigdb/cards/GO_PHAGOCYTOSIS) | 2.11e-5 | 3.31e-3 | **AXL**, TGFB1, F2RL1, EIF2AK1, **ELMO1** |
| [**DENDRITIC_CELL_DIFFERENTIATION**](http://www.gsea-msigdb.org/gsea/msigdb/cards/GO_DENDRITIC_CELL_DIFFERENTIATION) | 2.21e-5 | 3.39e-3 | **AXL**, TGFB1, F2RL1 |
| [RESPONSE_TO_ORGANIC_CYCLIC_COMPOUND](http://www.gsea-msigdb.org/gsea/msigdb/cards/GO_RESPONSE_TO_ORGANIC_CYCLIC_COMPOUND) | 2.26e-5 | 3.39e-3 | TGFB1, IFNA21, IFNA16, IFNA17, IFNA14, IFNA5, IFNA6, IFNA2 |
| [RESPONSE_TO_NITROGEN_COMPOUND](http://www.gsea-msigdb.org/gsea/msigdb/cards/GO_RESPONSE_TO_NITROGEN_COMPOUND) | 3.21e-5 | 4.72e-3 | TGFB1, IFNA21, IFNA16, IFNA17, IFNA14, IFNA5, IFNA6, IFNA2 |
| [**NEGATIVE_REGULATION_OF_SKELETAL_MUSCLE_TISSUE_DEVELOPMENT**](http://www.gsea-msigdb.org/gsea/msigdb/cards/GO_NEGATIVE_REGULATION_OF_SKELETAL_MUSCLE_TISSUE_DEVELOPMENT) | 1.25e-4 | 1.81e-2 | TGFB1, **EPHB1** |
| [REGULATION_OF_CYTOKINE_PRODUCTION_INVOLVED_IN_IMMUNE_RESPONSE](http://www.gsea-msigdb.org/gsea/msigdb/cards/GO_REGULATION_OF_CYTOKINE_PRODUCTION_INVOLVED_IN_IMMUNE_RESPONSE) | 1.77e-4 | 2.51e-2 | TGFB1, F2RL1, IFNA2 |
| [POSITIVE_REGULATION_OF_PROTEIN_METABOLIC_PROCESS](http://www.gsea-msigdb.org/gsea/msigdb/cards/GO_POSITIVE_REGULATION_OF_PROTEIN_METABOLIC_PROCESS) | 1.94e-4 | 2.70e-2 | TGFB1, F2RL1, IFNA21, IFNA16, IFNA17, IFNA14, IFNA5, IFNA6, IFNA2 |
| [POSITIVE_REGULATION_OF_SUPEROXIDE_ANION_GENERATION](http://www.gsea-msigdb.org/gsea/msigdb/cards/GO_POSITIVE_REGULATION_OF_SUPEROXIDE_ANION_GENERATION) | 2.27e-4 | 3.09e-2 | TGFB1, F2RL1 |
| [CYTOKINE_PRODUCTION_INVOLVED_IN_IMMUNE_RESPONSE](http://www.gsea-msigdb.org/gsea/msigdb/cards/GO_CYTOKINE_PRODUCTION_INVOLVED_IN_IMMUNE_RESPONSE) | 3.18e-4 | 4.24e-2 | TGFB1, F2RL1, IFNA2 |
| [ARF_PROTEIN_SIGNAL_TRANSDUCTION](http://www.gsea-msigdb.org/gsea/msigdb/cards/GO_ARF_PROTEIN_SIGNAL_TRANSDUCTION) | 3.23e-4 | 4.24e-2 | CYTH1, CYTH4 |
| [REGULATION_OF_SUPEROXIDE_ANION_GENERATION](http://www.gsea-msigdb.org/gsea/msigdb/cards/GO_REGULATION_OF_SUPEROXIDE_ANION_GENERATION) | 3.58e-4 | 4.62e-2 | TGFB1, F2RL1 |

Only significant terms are described. Genesets which contain genes that were significant in our study are highlighted in bold.

[**Gene Ontology Molecular Functions**](https://fuma.ctglab.nl/gene2func/95220#GO_mfPanel)

| **GeneSet** | ***P*** | **Adjusted *P*** | **Genes** |
| --- | --- | --- | --- |
| [TYPE_I_INTERFERON_RECEPTOR_BINDING](http://www.gsea-msigdb.org/gsea/msigdb/cards/GO_TYPE_I_INTERFERON_RECEPTOR_BINDING) | 9.17e-17 | 1.51e-13 | IFNA21, IFNA16, IFNA17, IFNA14, IFNA5, IFNA6, IFNA2 |
| [CYTOKINE_ACTIVITY](http://www.gsea-msigdb.org/gsea/msigdb/cards/GO_CYTOKINE_ACTIVITY) | 4.11e-10 | 3.38e-7 | TGFB1, IFNA21, IFNA16, IFNA17, IFNA14, IFNA5, IFNA6, IFNA2 |
| [CYTOKINE_RECEPTOR_BINDING](http://www.gsea-msigdb.org/gsea/msigdb/cards/GO_CYTOKINE_RECEPTOR_BINDING) | 3.13e-9 | 1.72e-6 | TGFB1, IFNA21, IFNA16, IFNA17, IFNA14, IFNA5, IFNA6, IFNA2 |
| [RECEPTOR_REGULATOR_ACTIVITY](http://www.gsea-msigdb.org/gsea/msigdb/cards/GO_RECEPTOR_REGULATOR_ACTIVITY) | 3.47e-7 | 1.43e-4 | TGFB1, IFNA21, IFNA16, IFNA17, IFNA14, IFNA5, IFNA6, IFNA2 |
| [**MOLECULAR_FUNCTION_REGULATOR**](http://www.gsea-msigdb.org/gsea/msigdb/cards/GO_MOLECULAR_FUNCTION_REGULATOR) | 1.16e-5 | 3.81e-3 | CYTH1, TGFB1, CYTH4, **ELMO1**, IFNA21, IFNA16, IFNA17, IFNA14, IFNA5, IFNA6, IFNA2 |
| [**PROTEIN_CONTAINING_COMPLEX_BINDING**](http://www.gsea-msigdb.org/gsea/msigdb/cards/GO_PROTEIN_CONTAINING_COMPLEX_BINDING) | 7.73e-5 | 2.12e-2 | **EPHB1**, IFNA21, IFNA16, IFNA17, IFNA14, IFNA5, IFNA6, IFNA2 |
| [SIGNALING_RECEPTOR_BINDING](http://www.gsea-msigdb.org/gsea/msigdb/cards/GO_SIGNALING_RECEPTOR_BINDING) | 1.55e-4 | 3.64e-2 | TGFB1, F2RL1, IFNA21, IFNA16, IFNA17, IFNA14, IFNA5, IFNA6, IFNA2 |

Only significant terms are described. Genesets which contain genes that were significant in our study are highlighted in bold.
